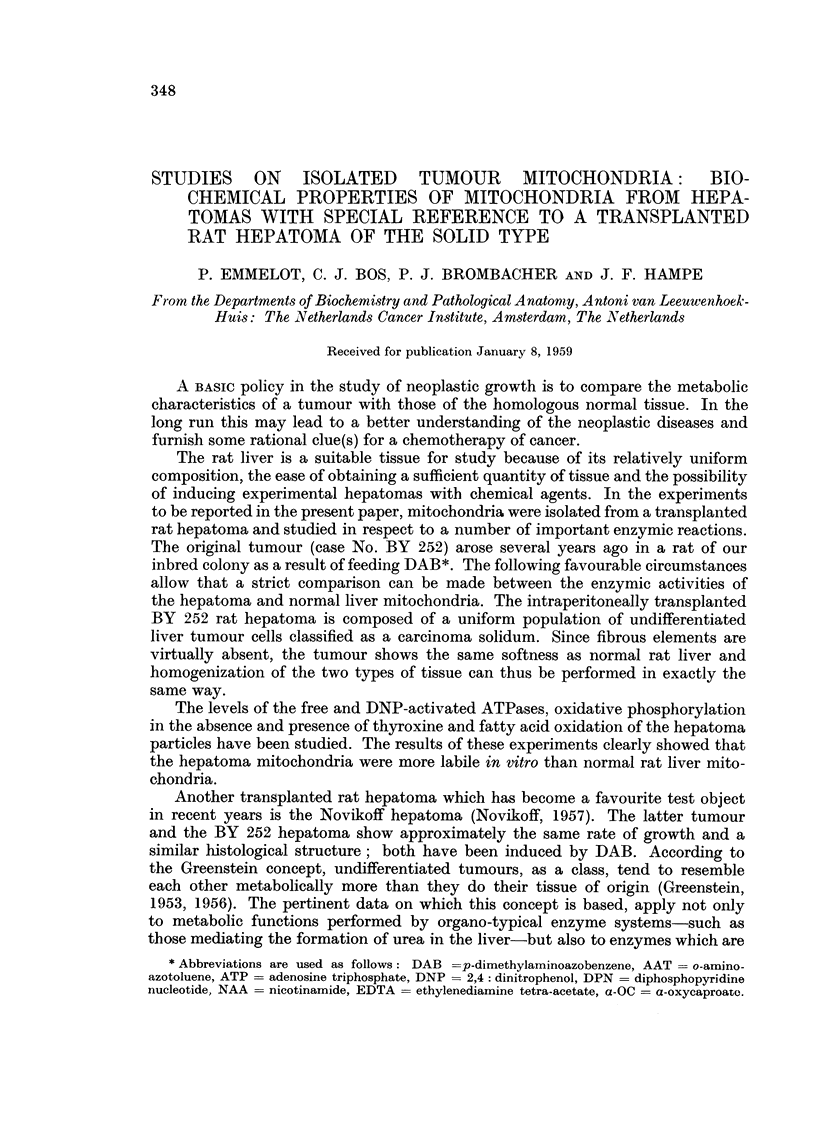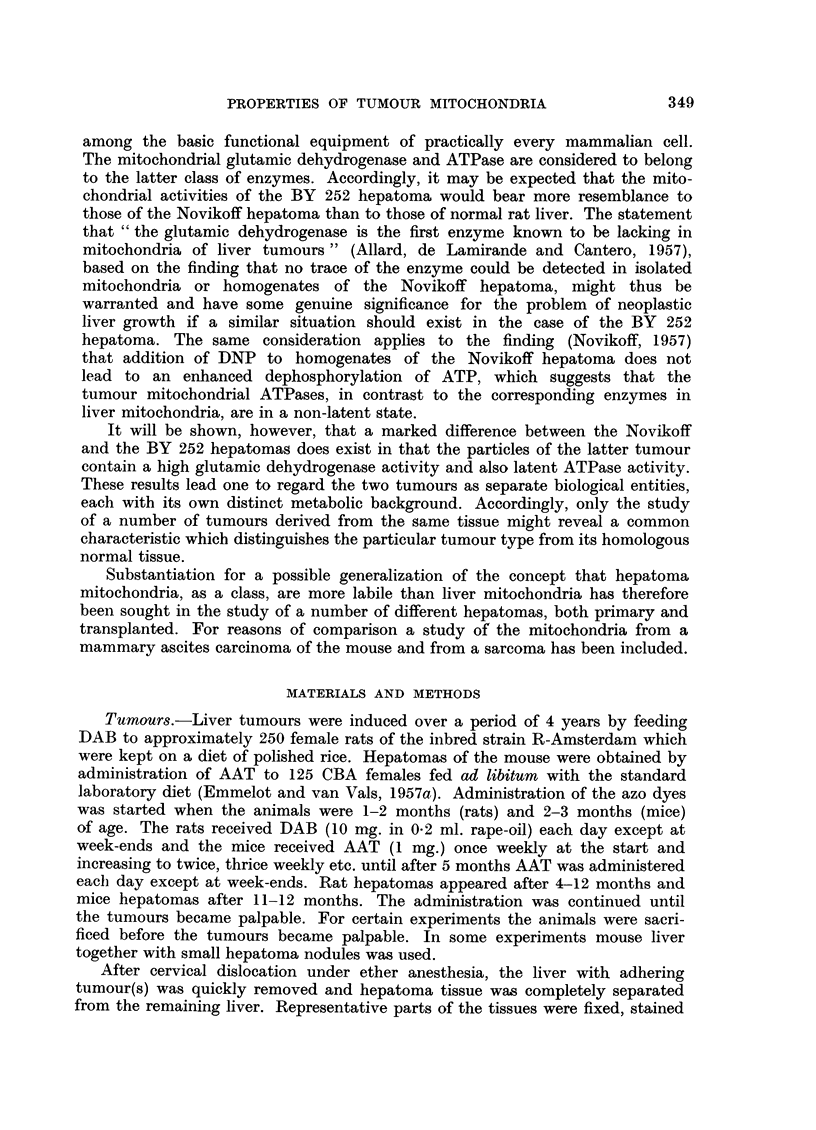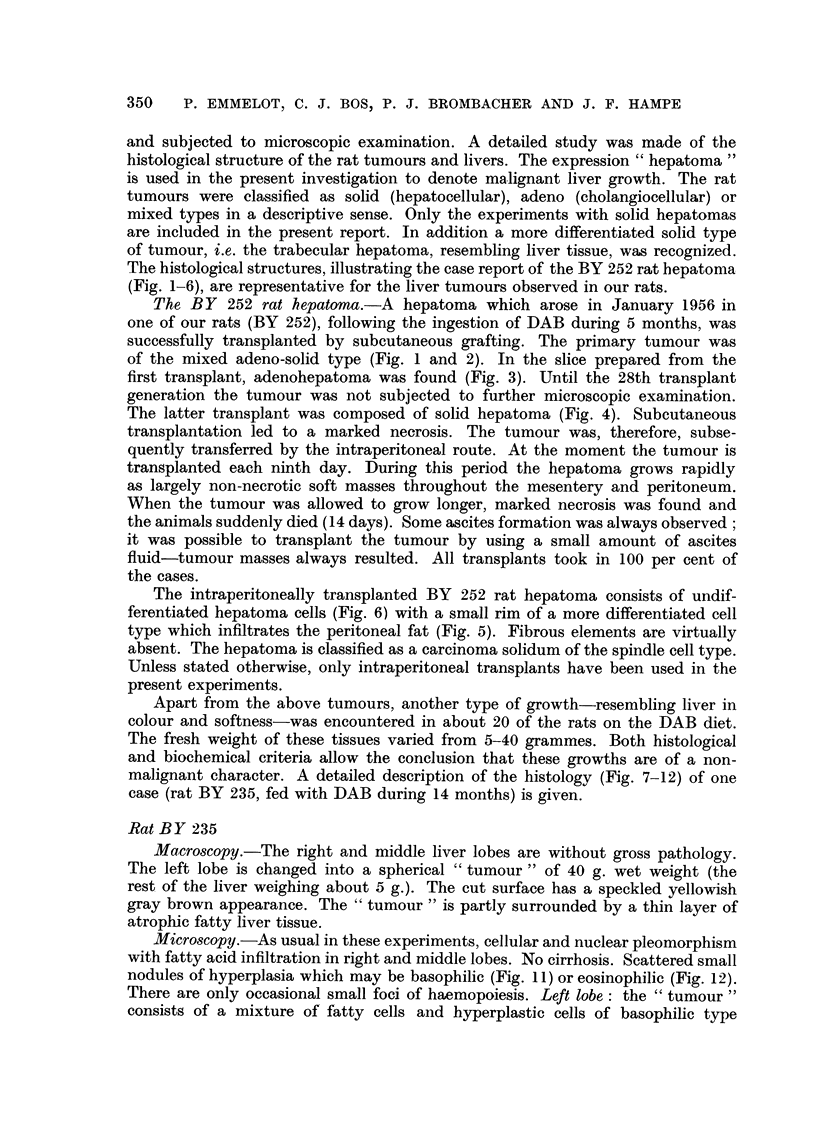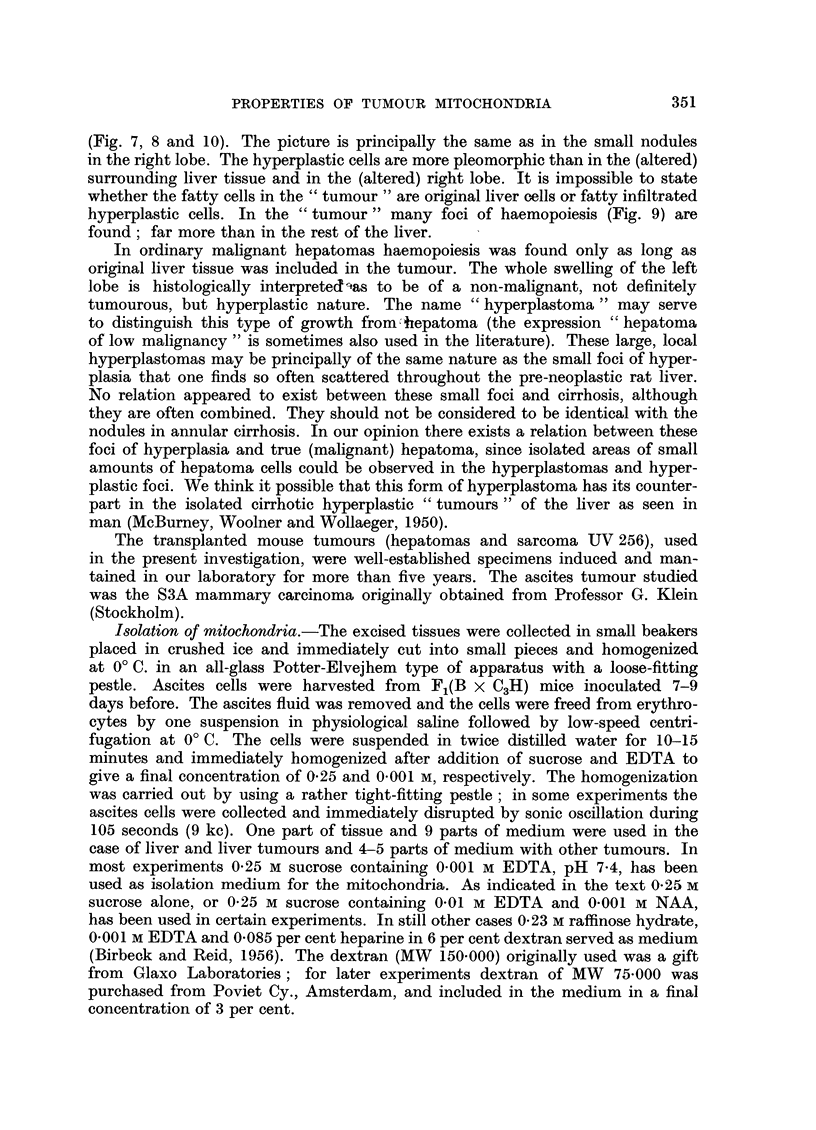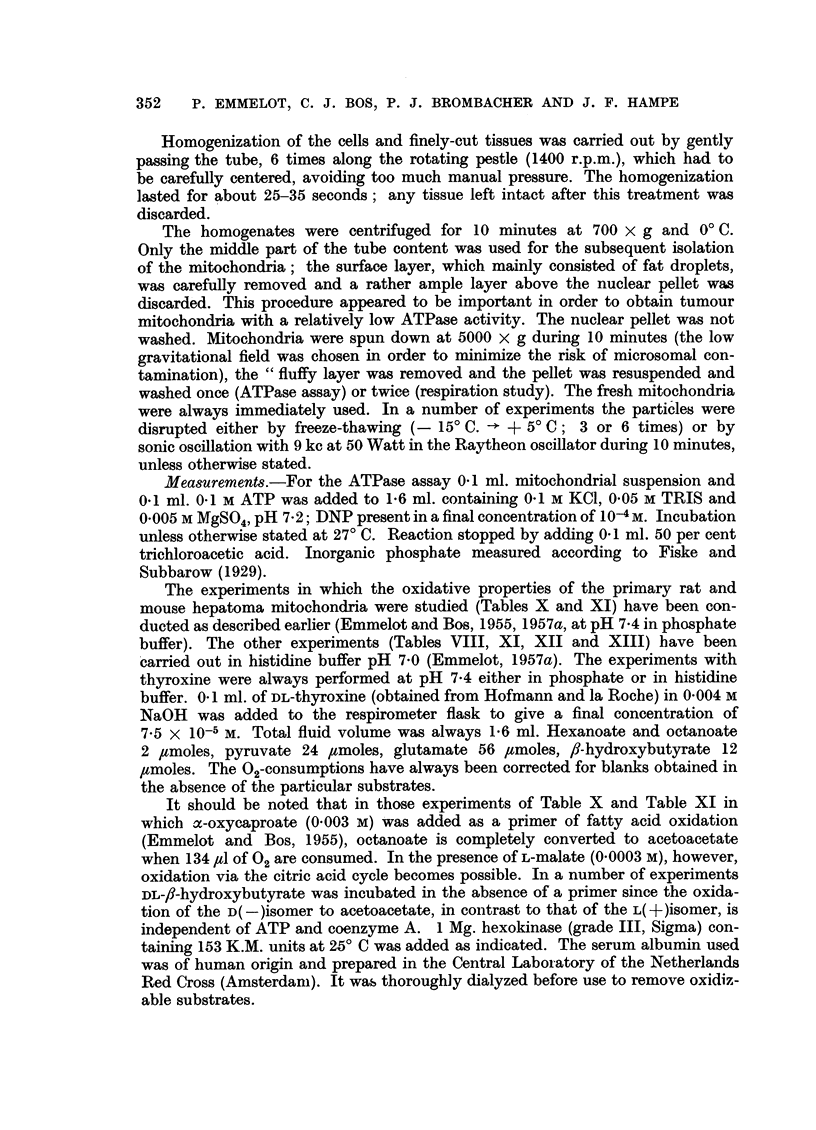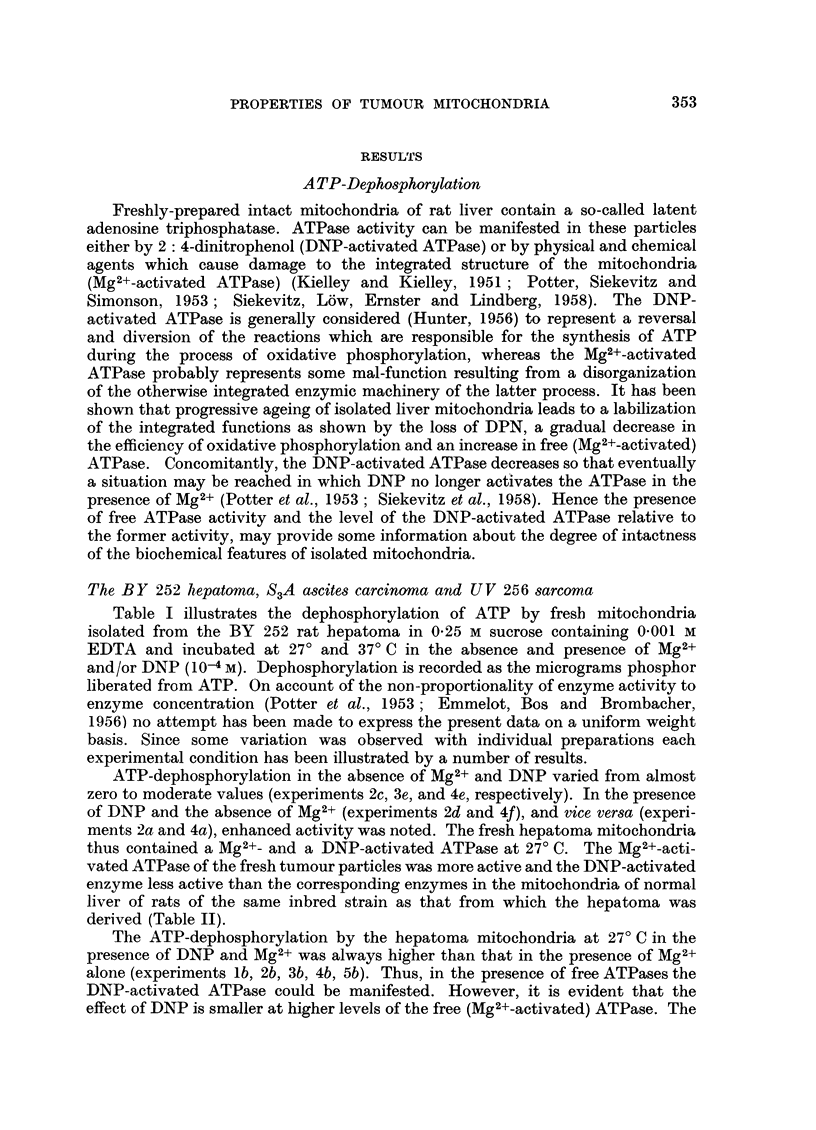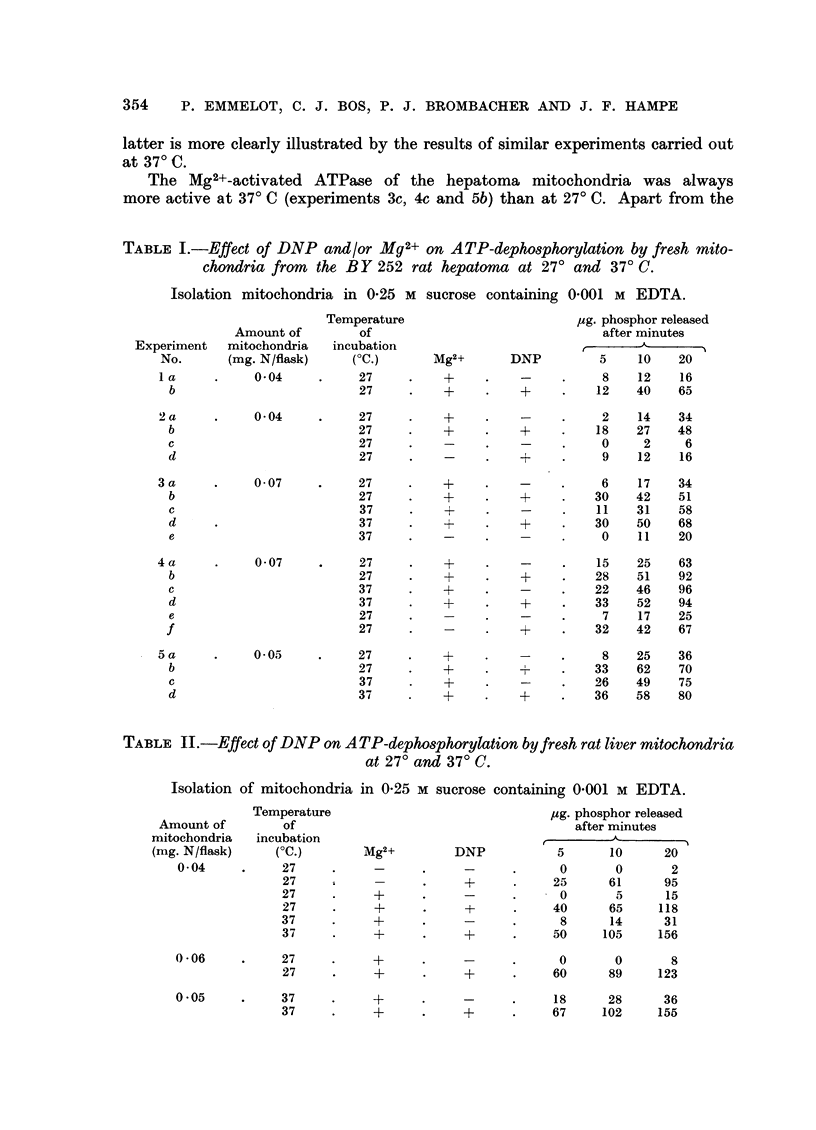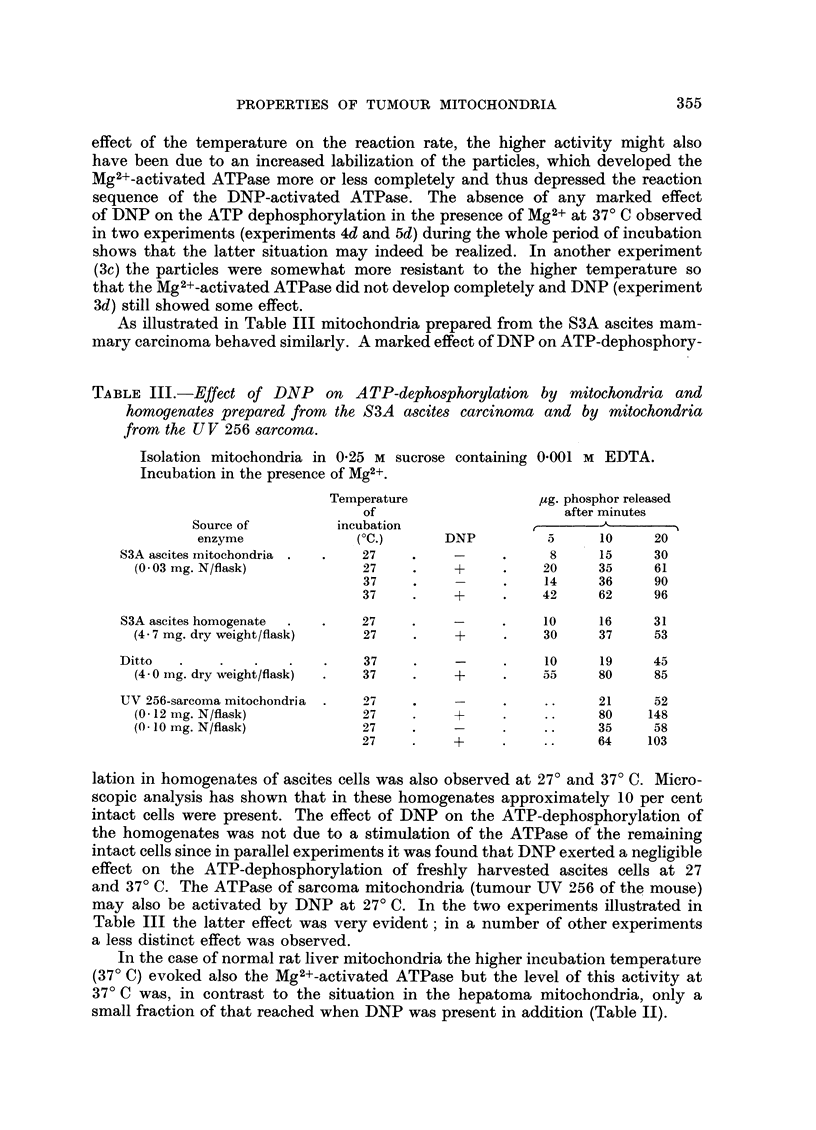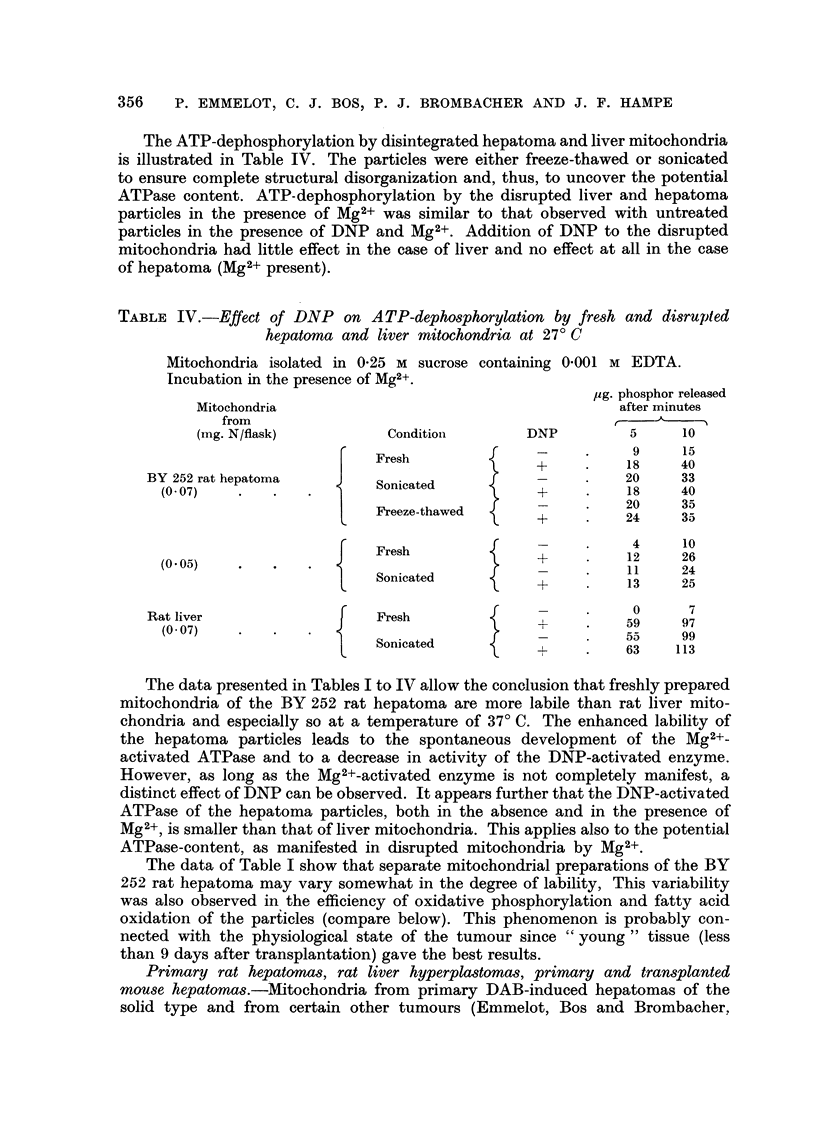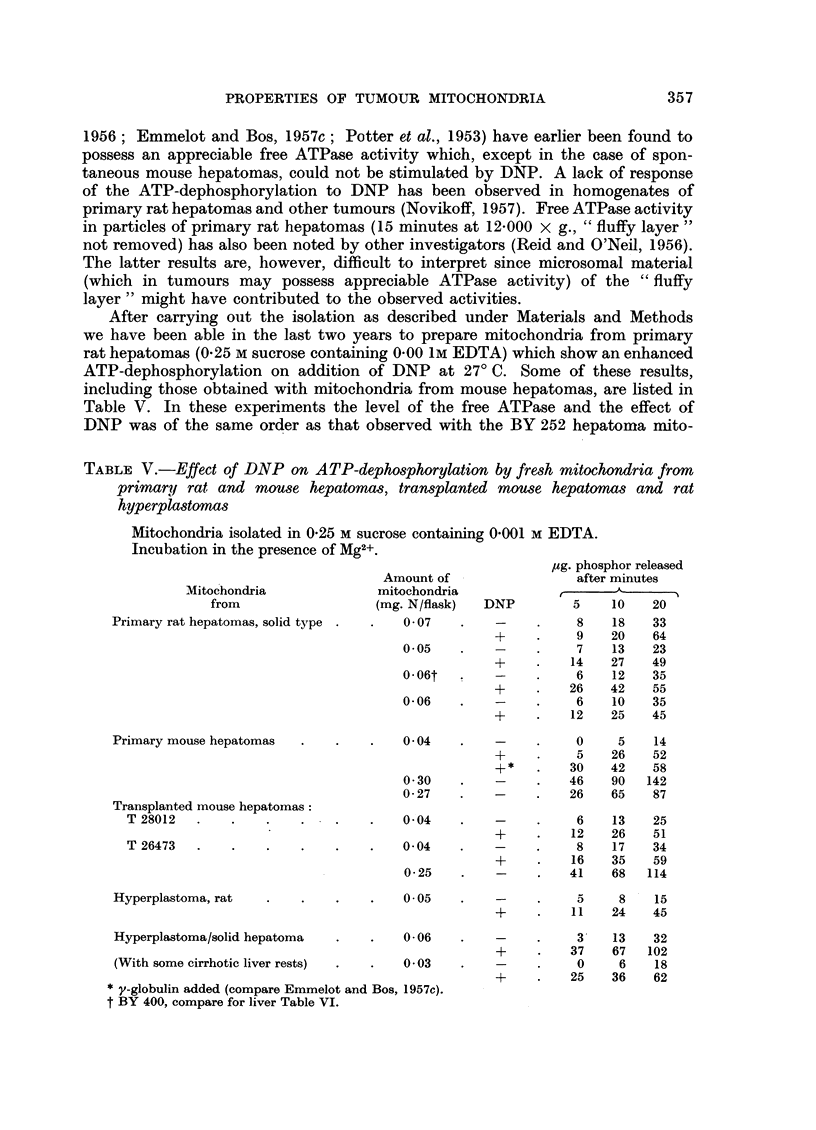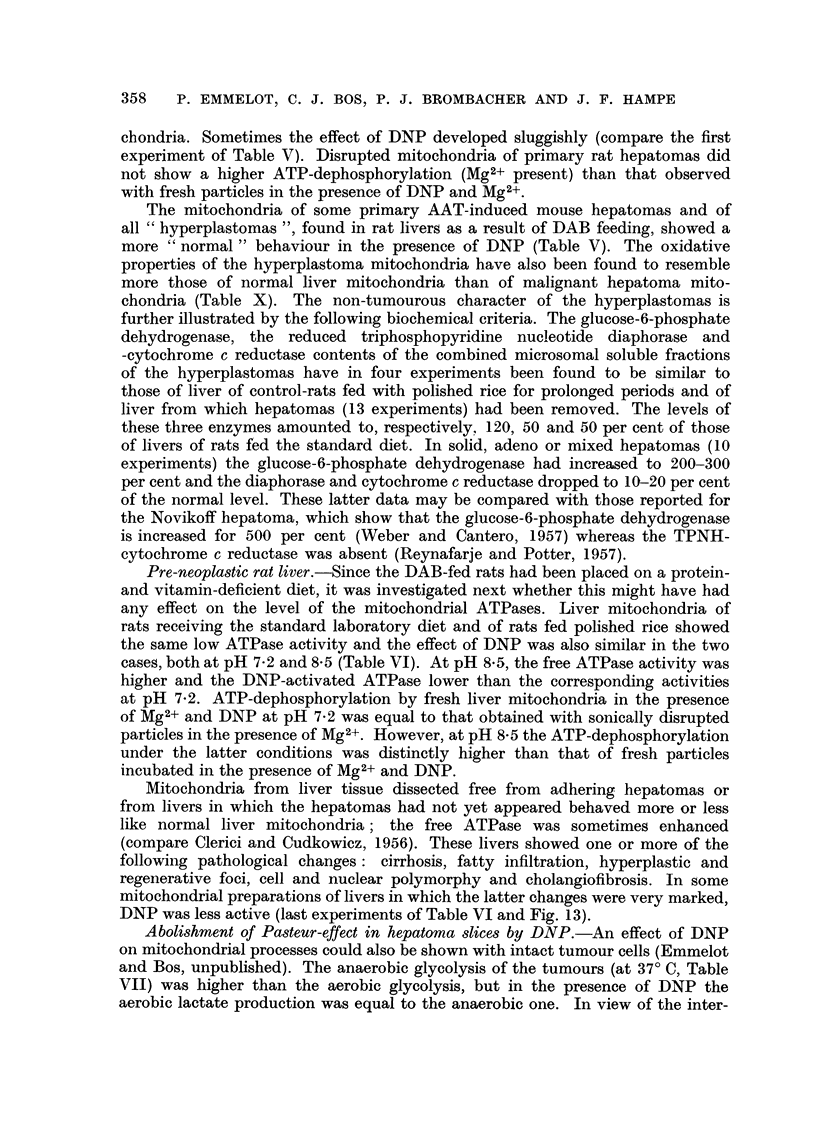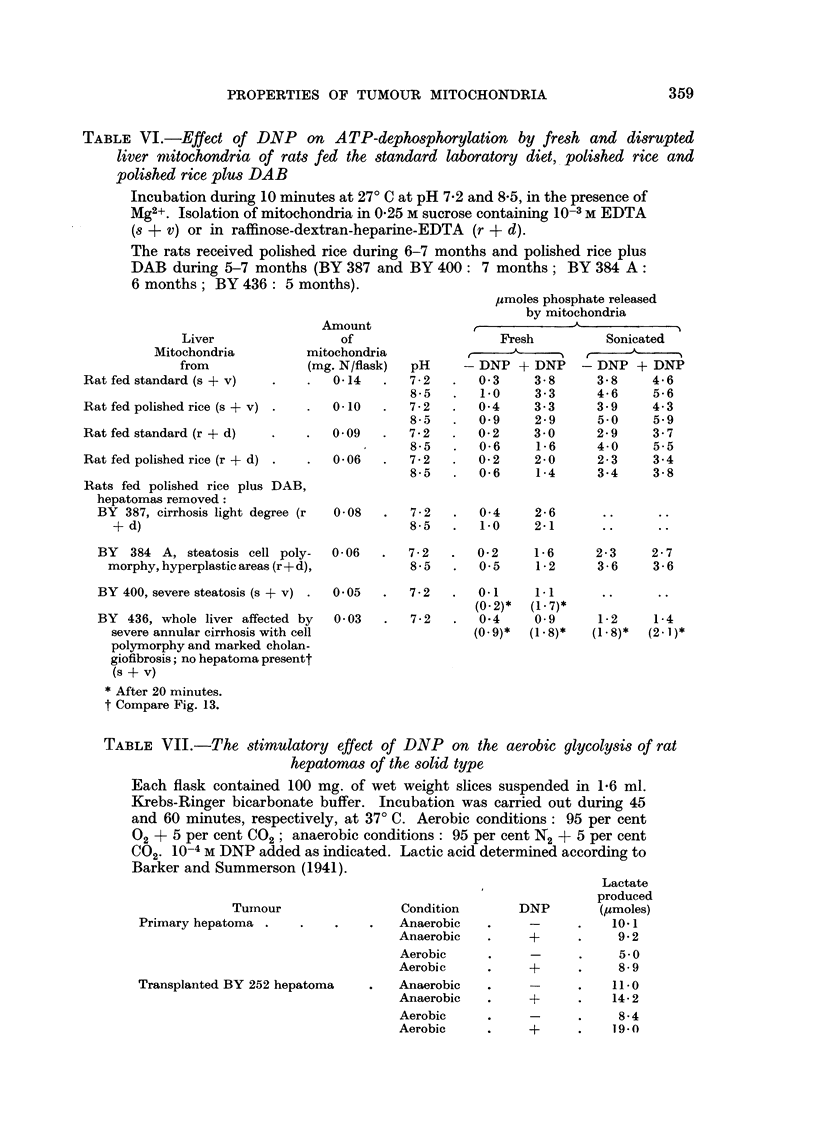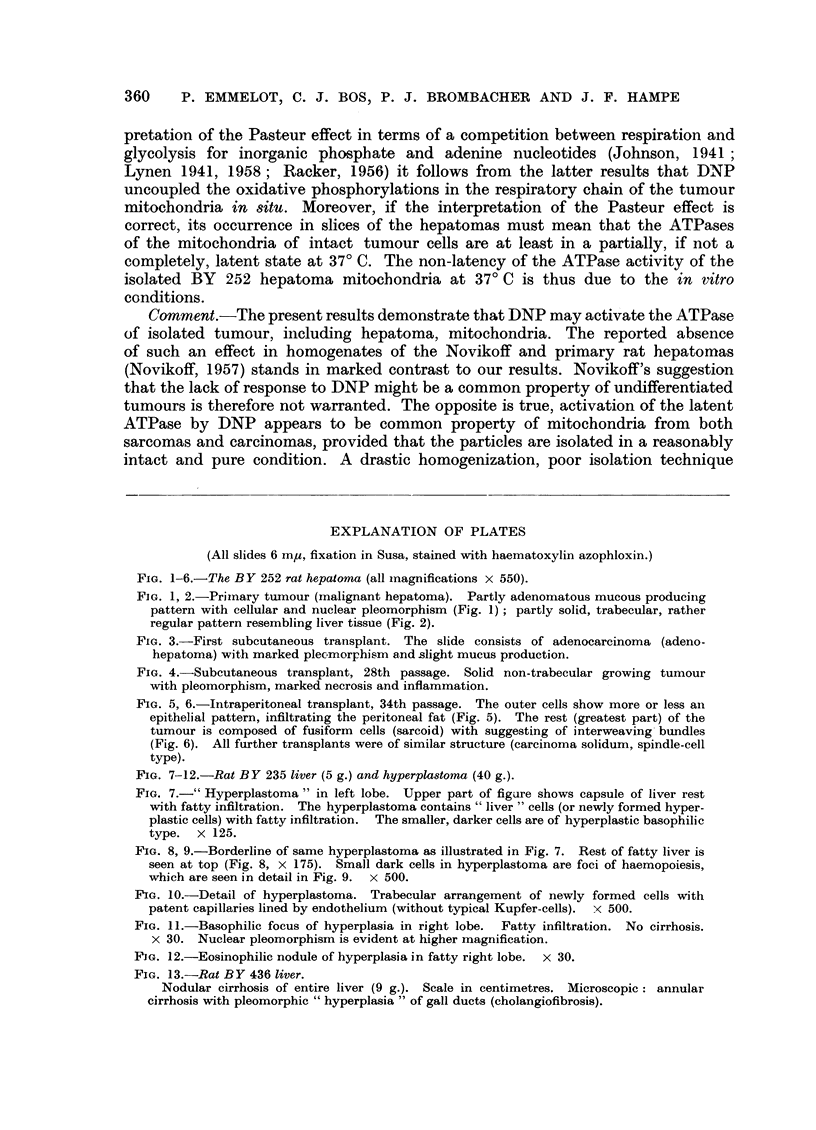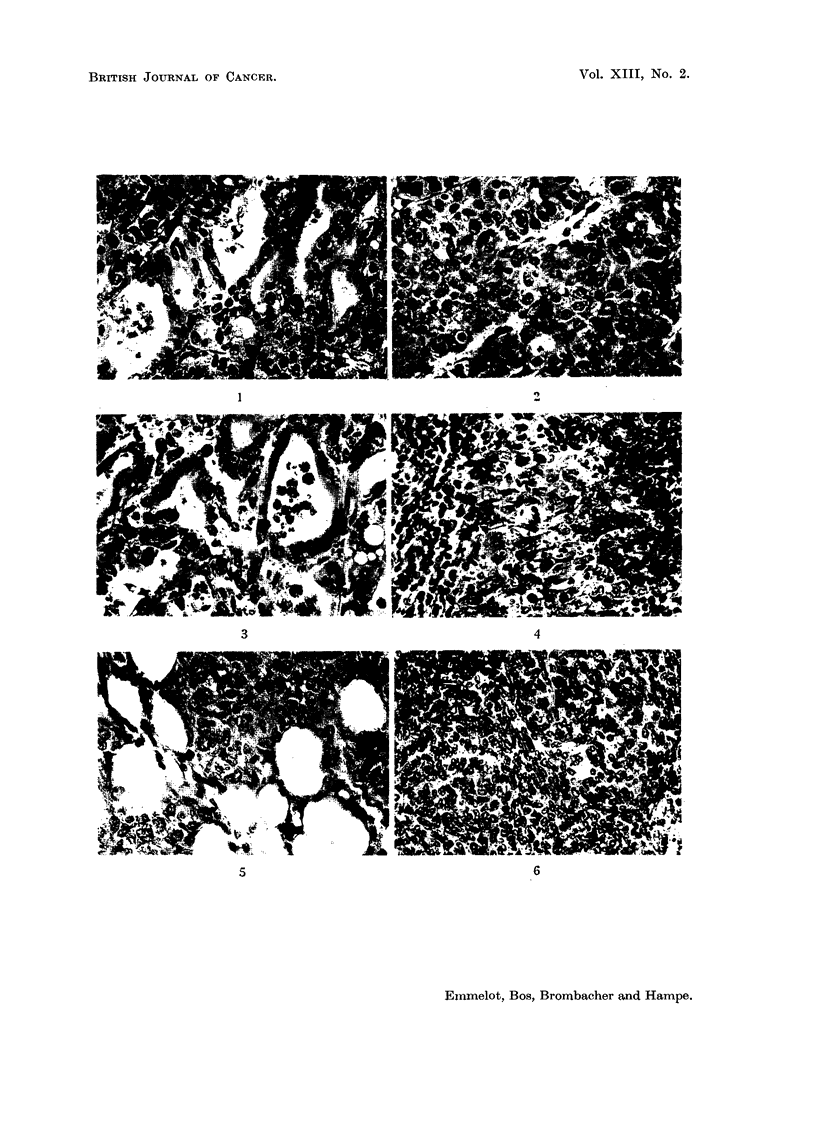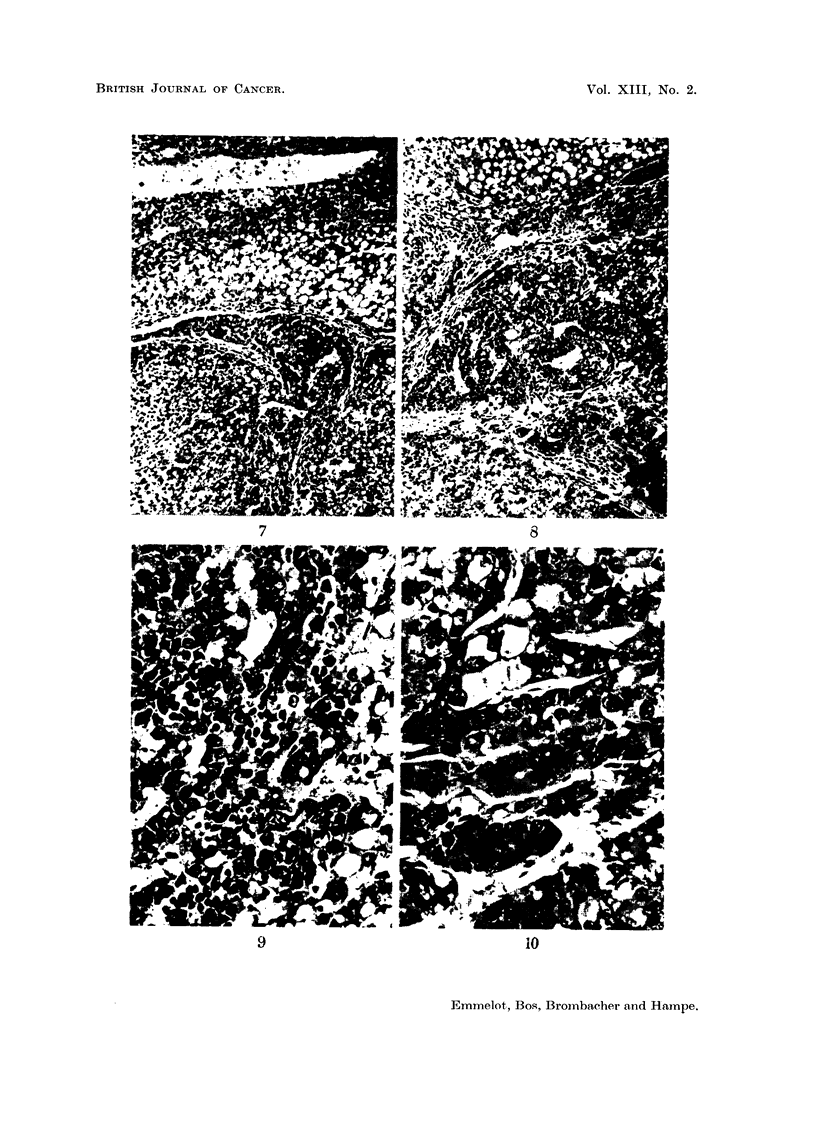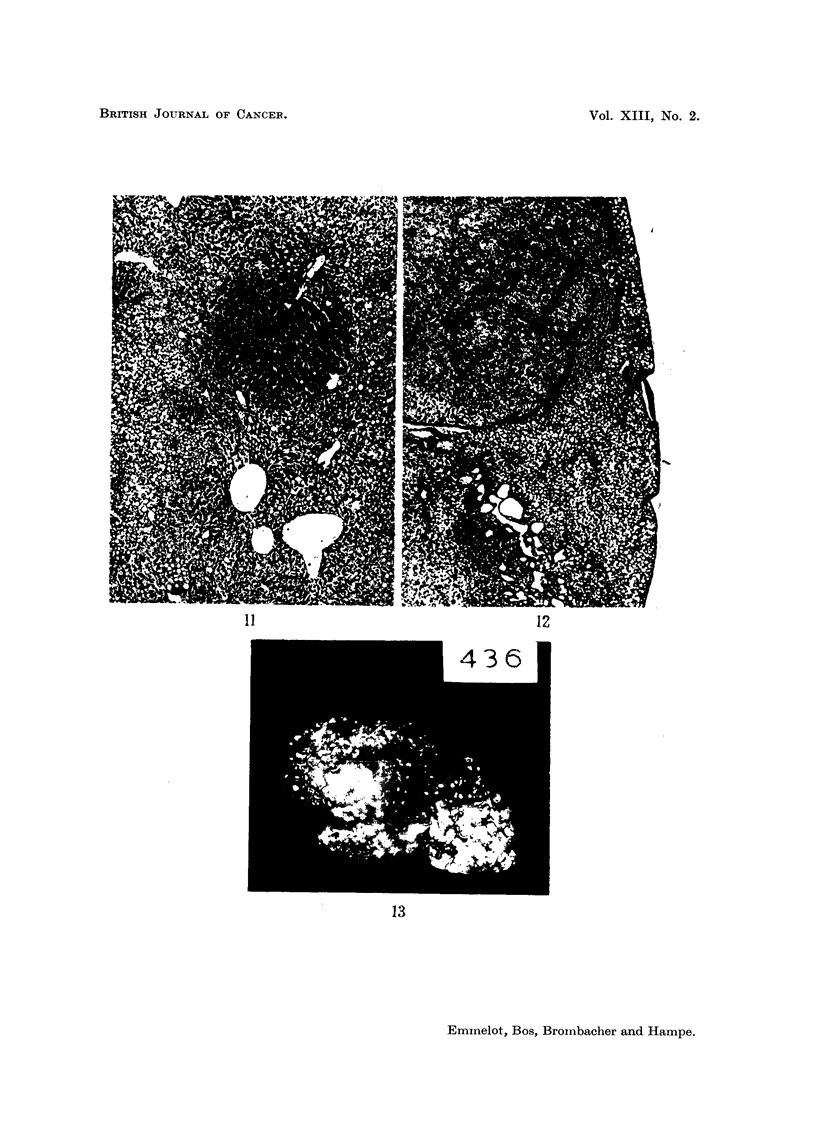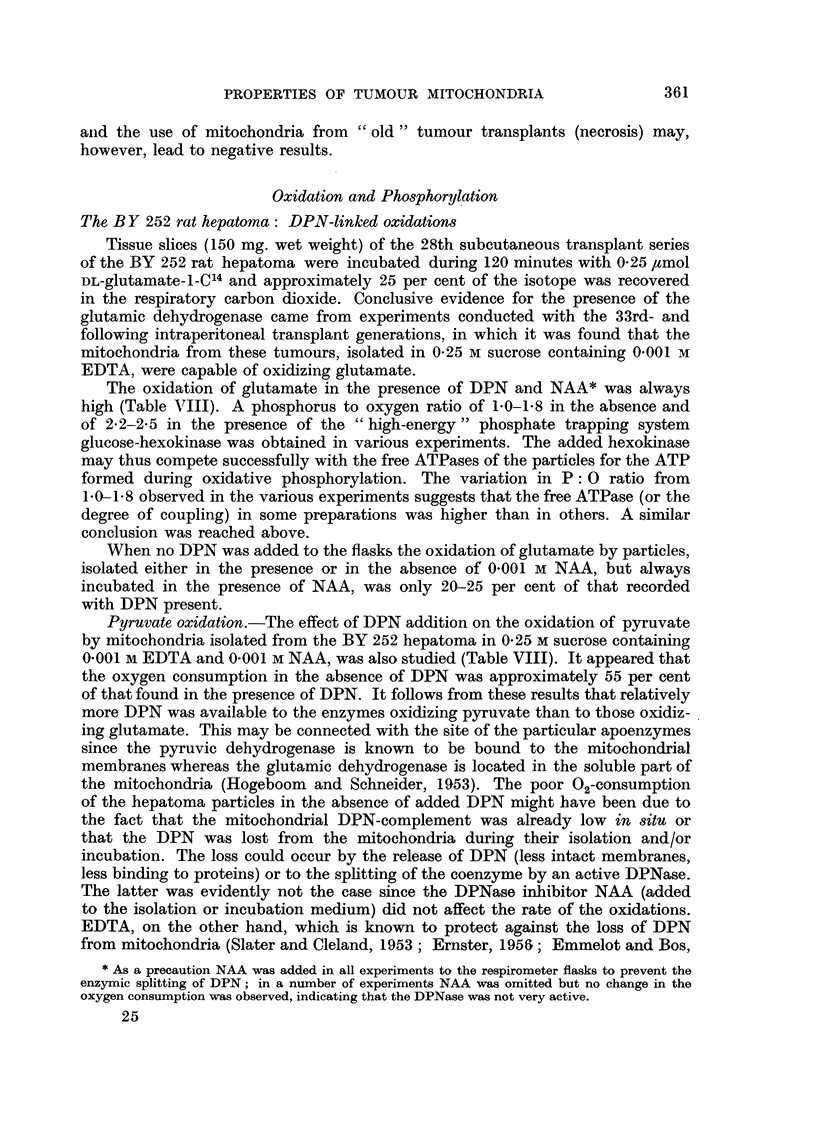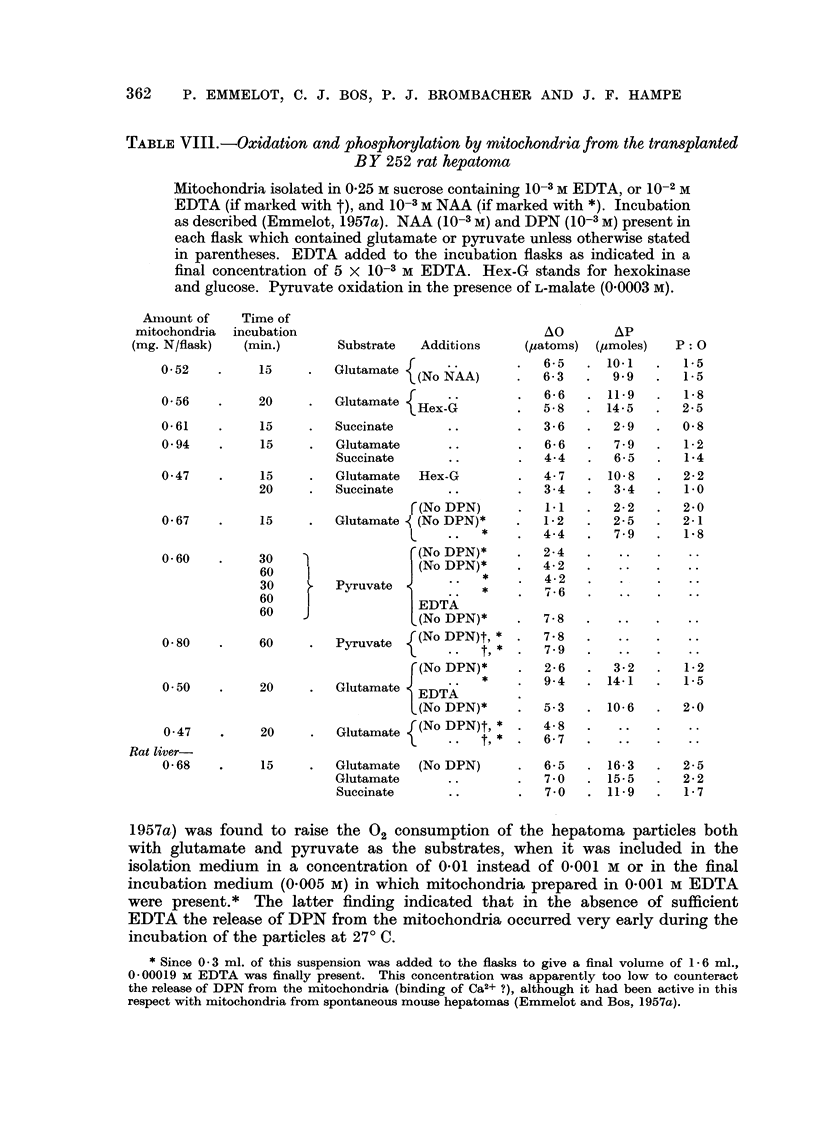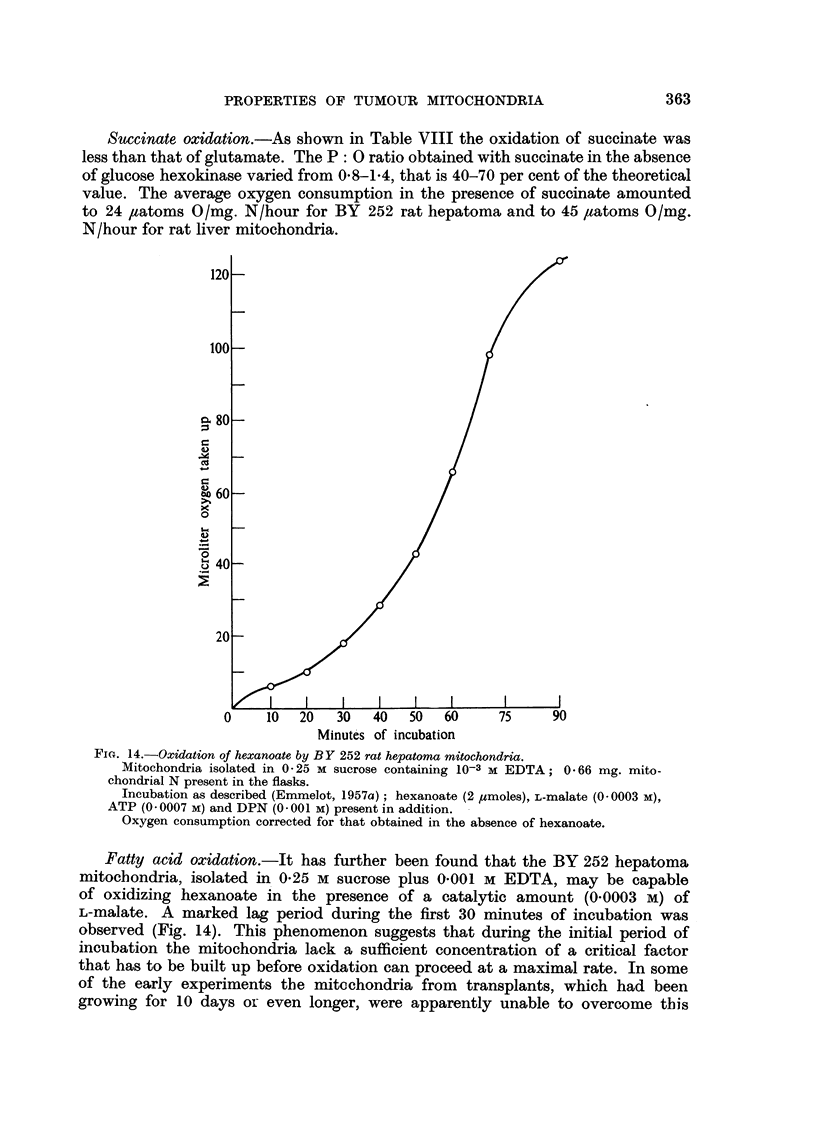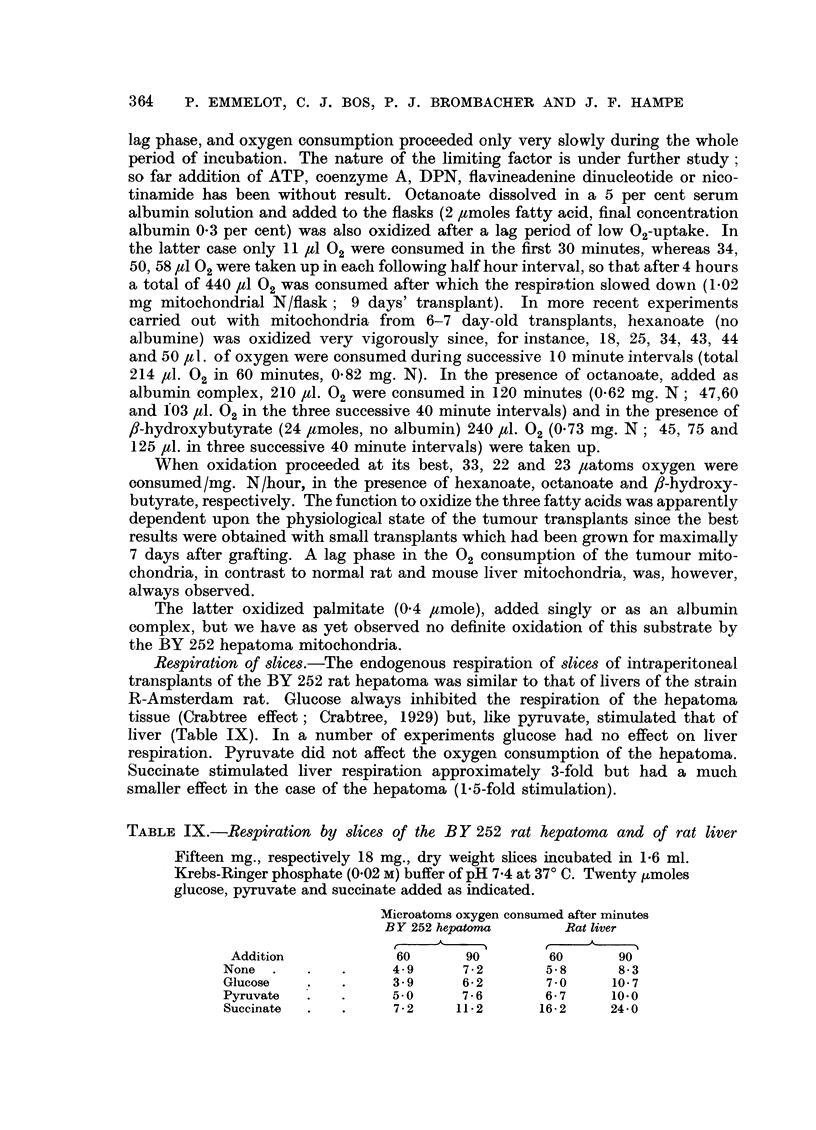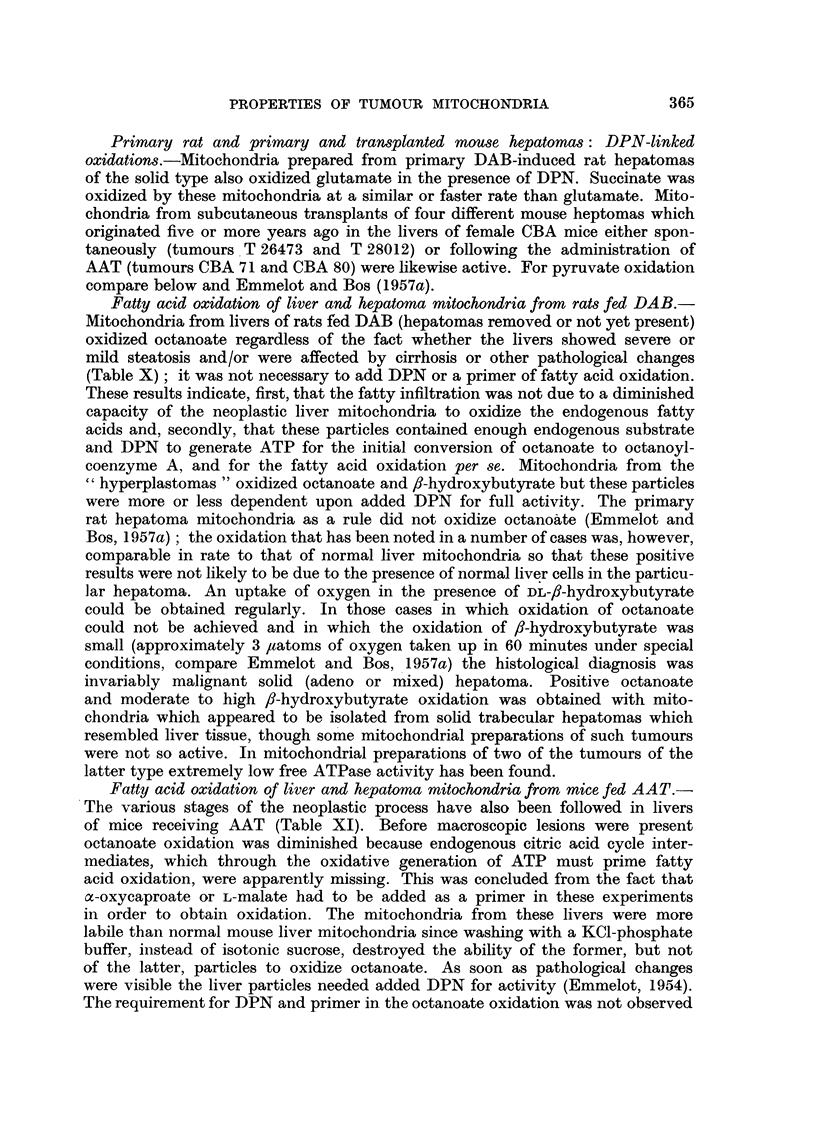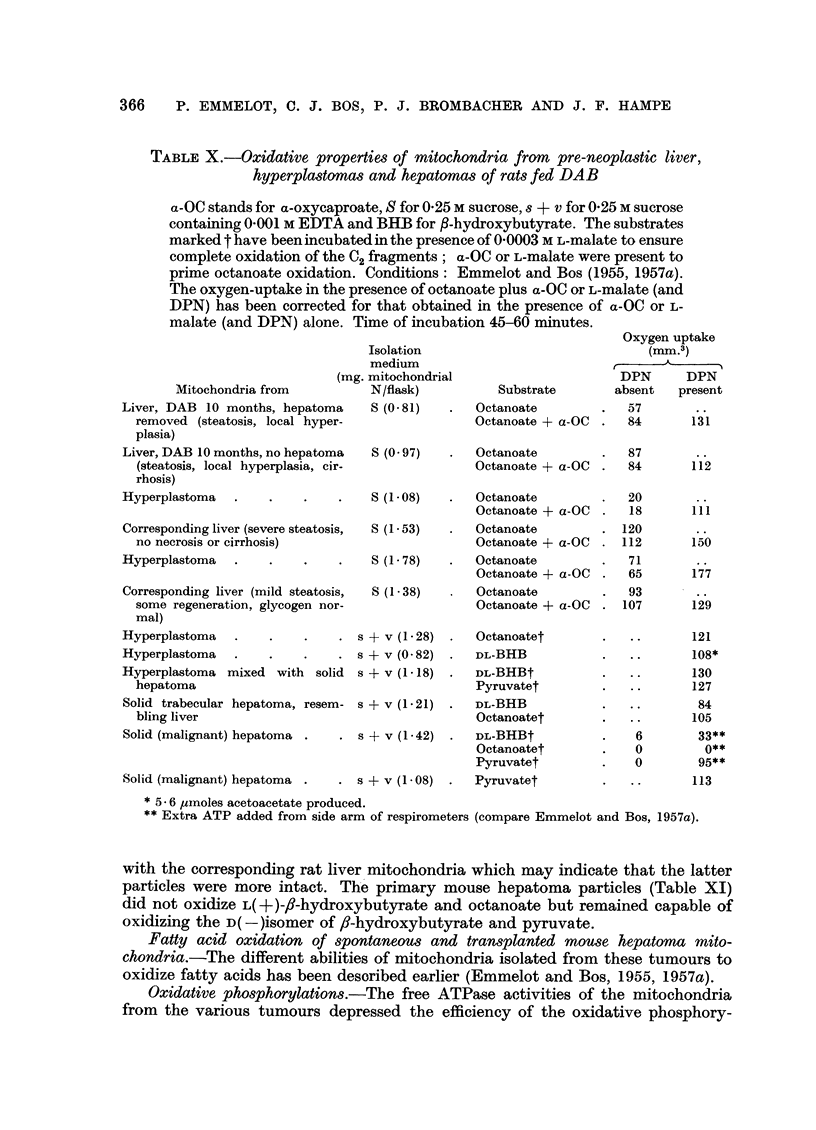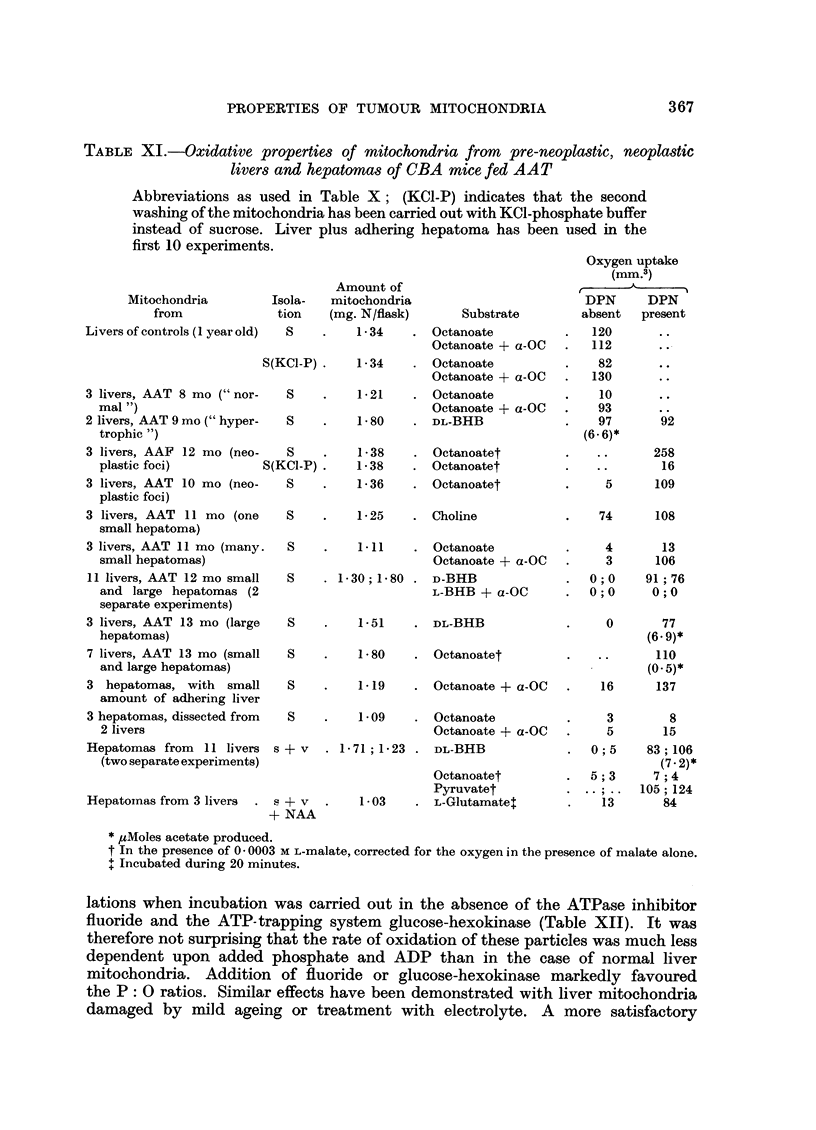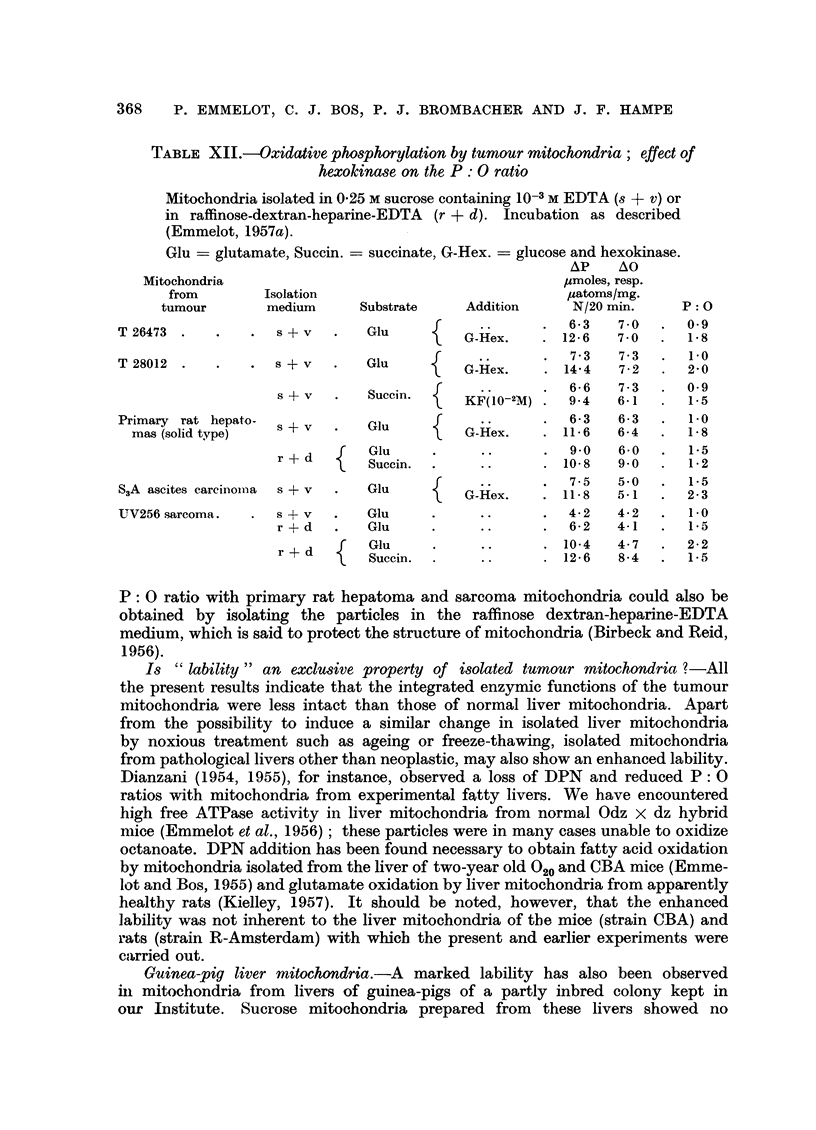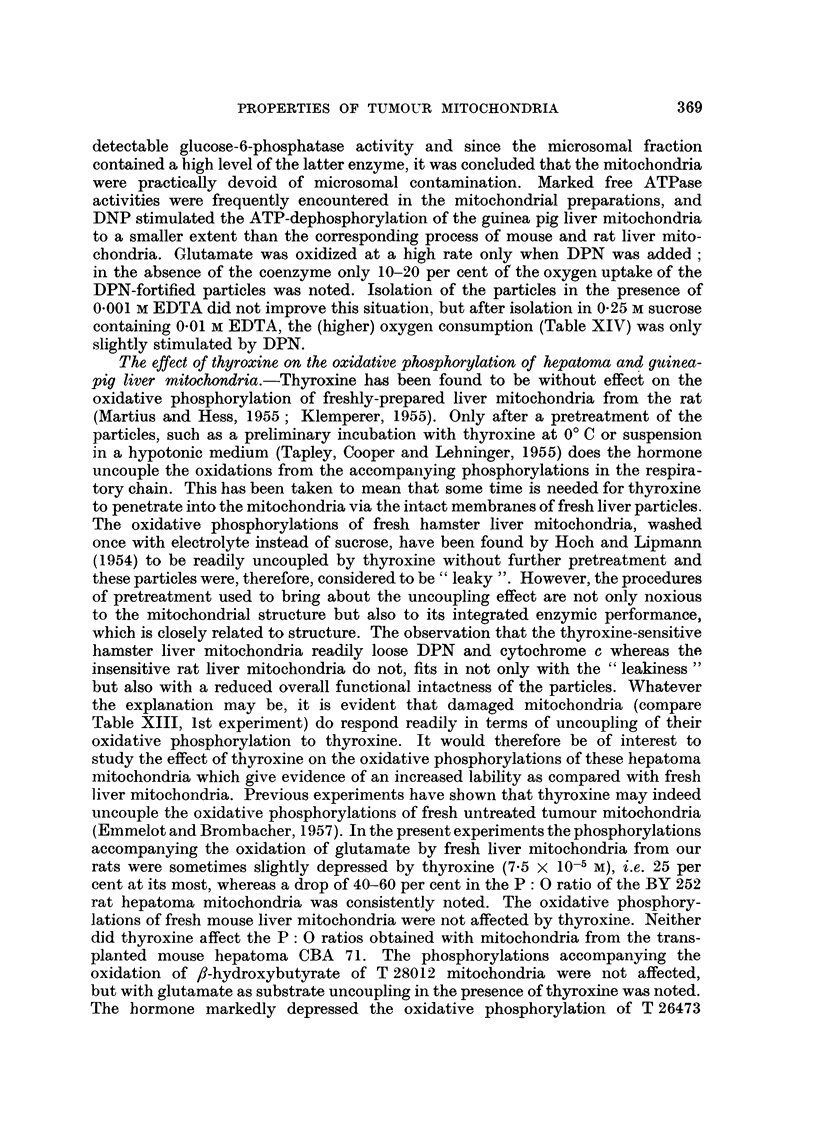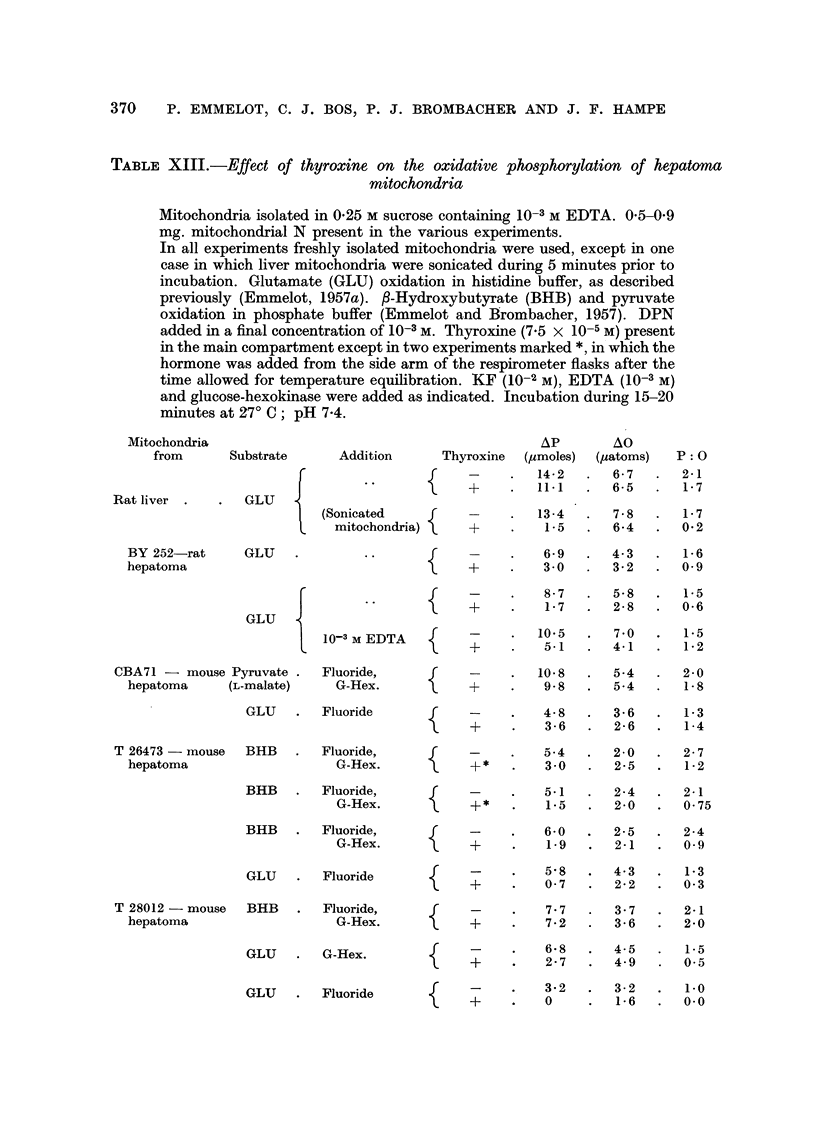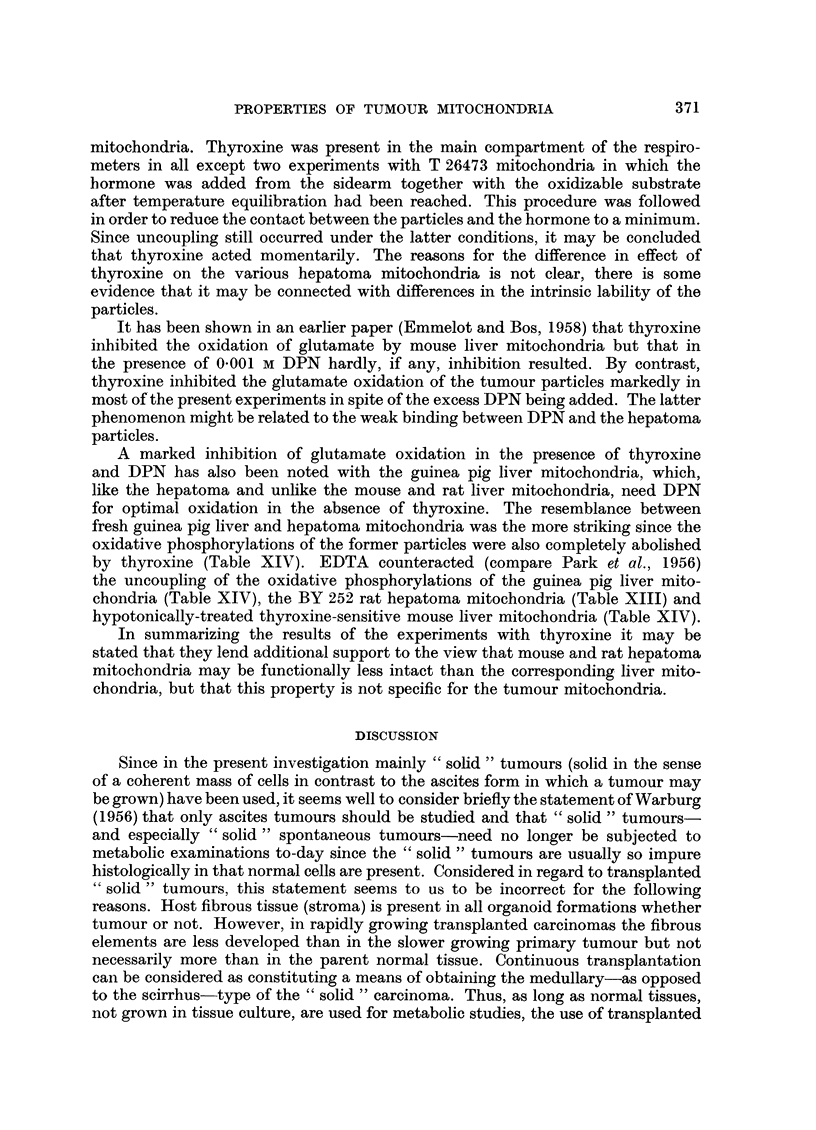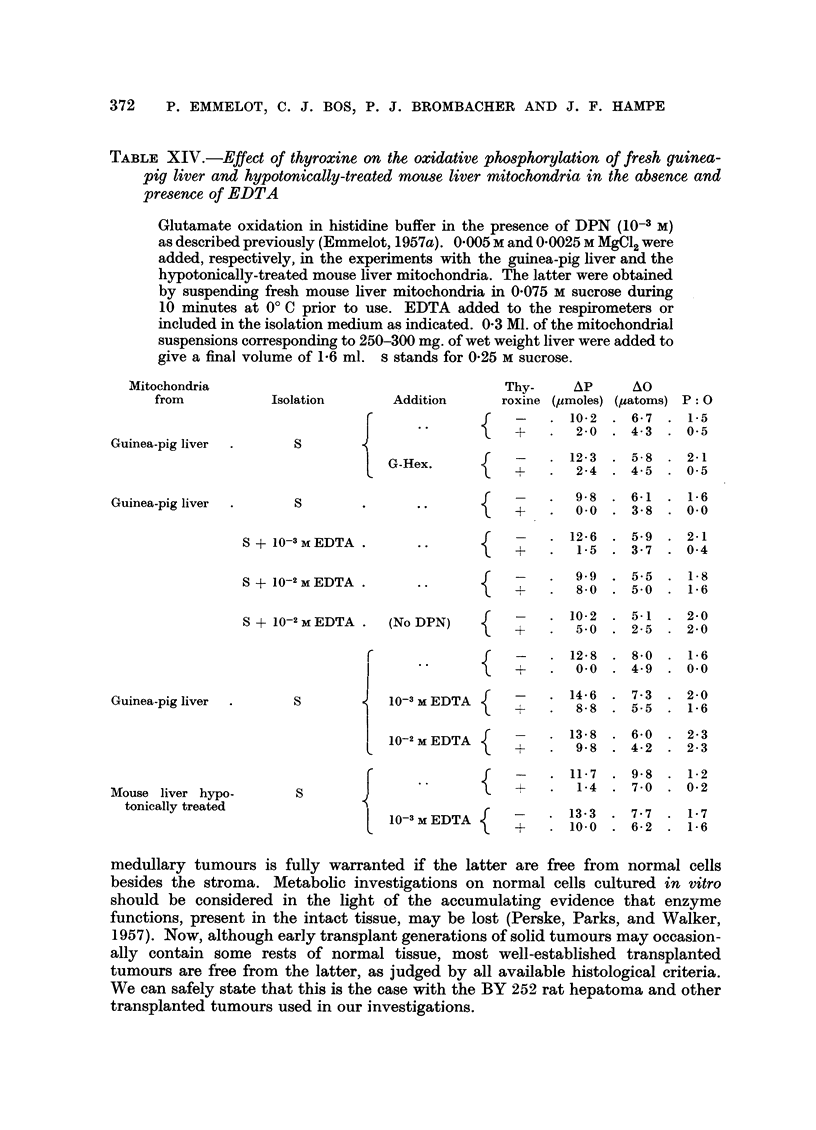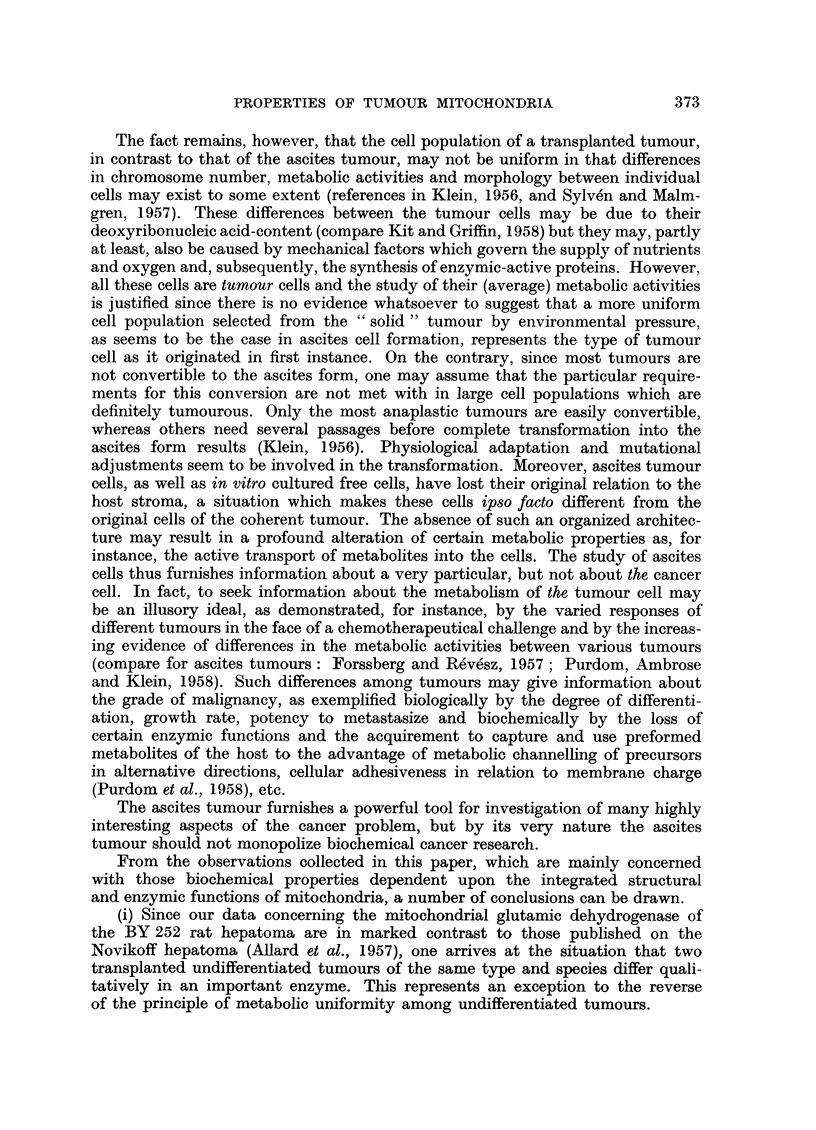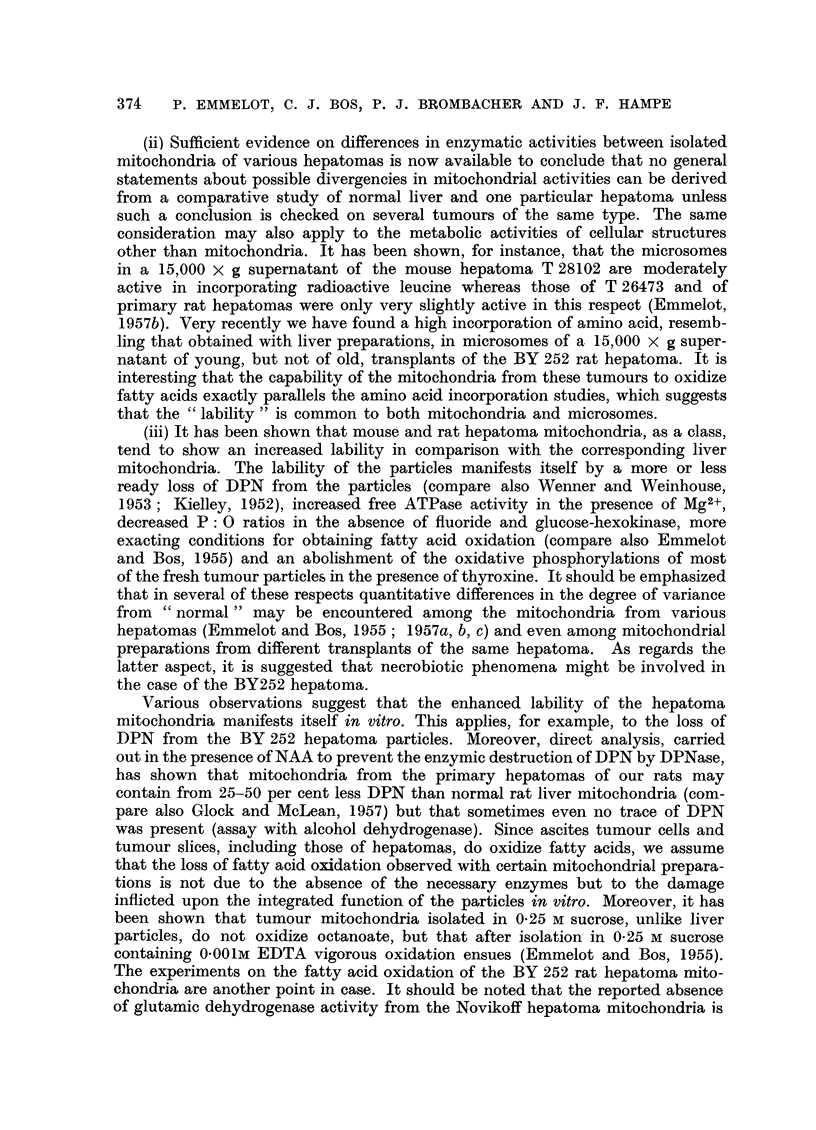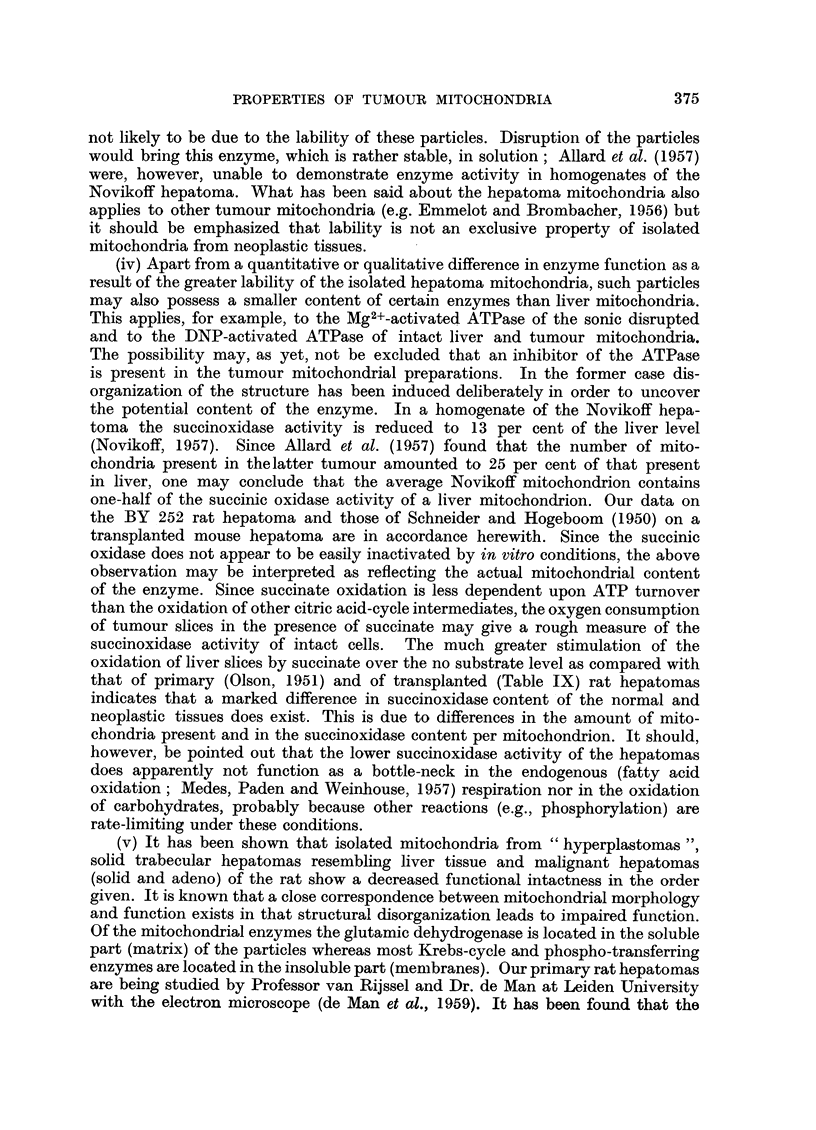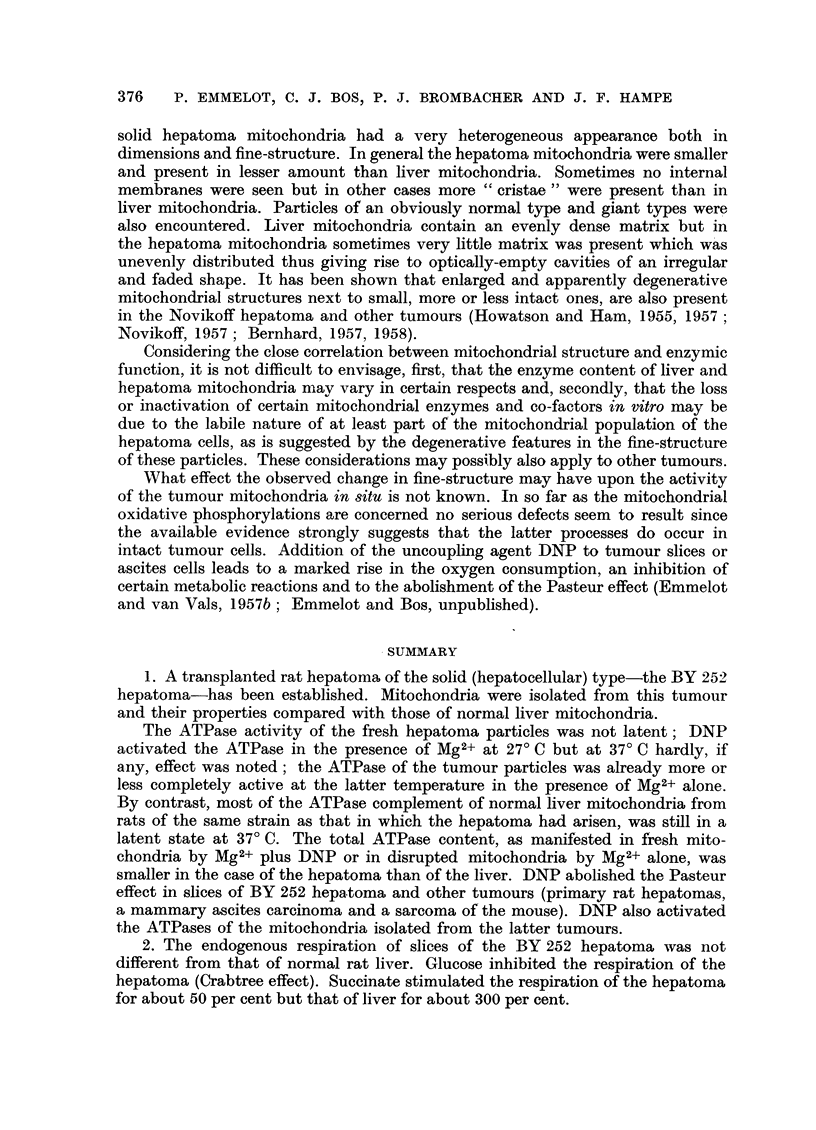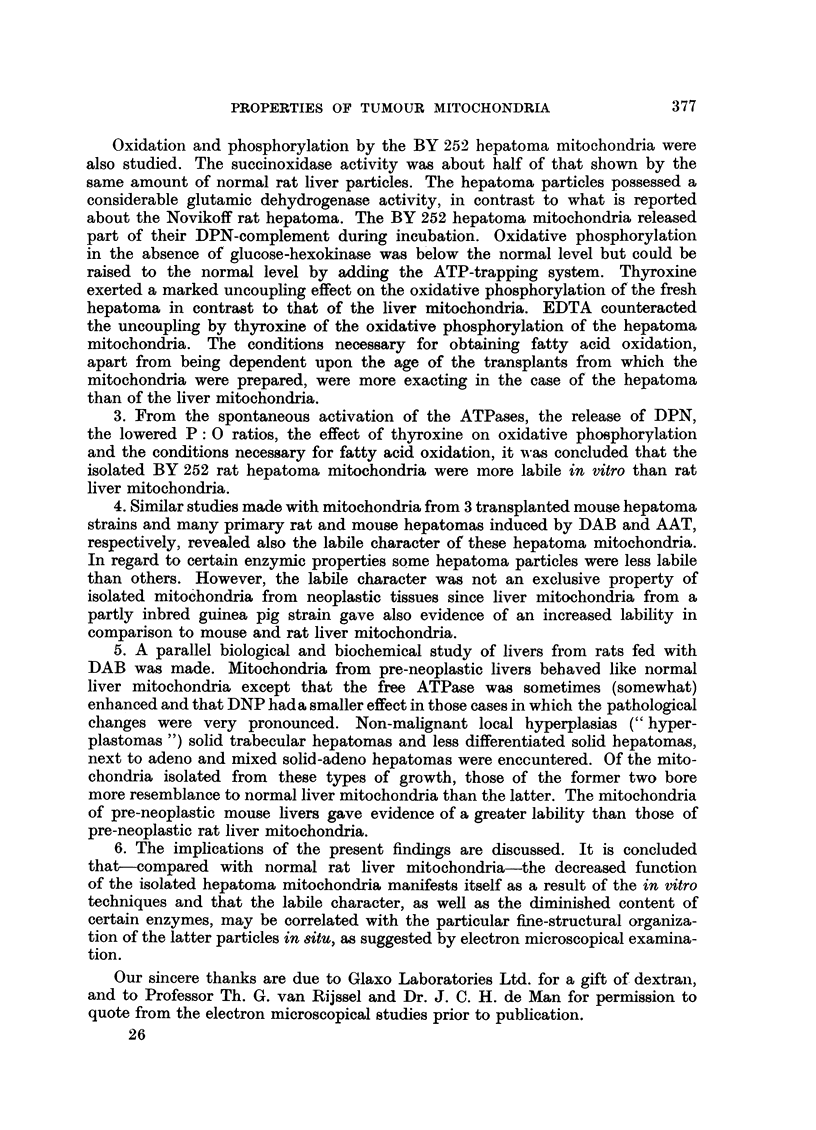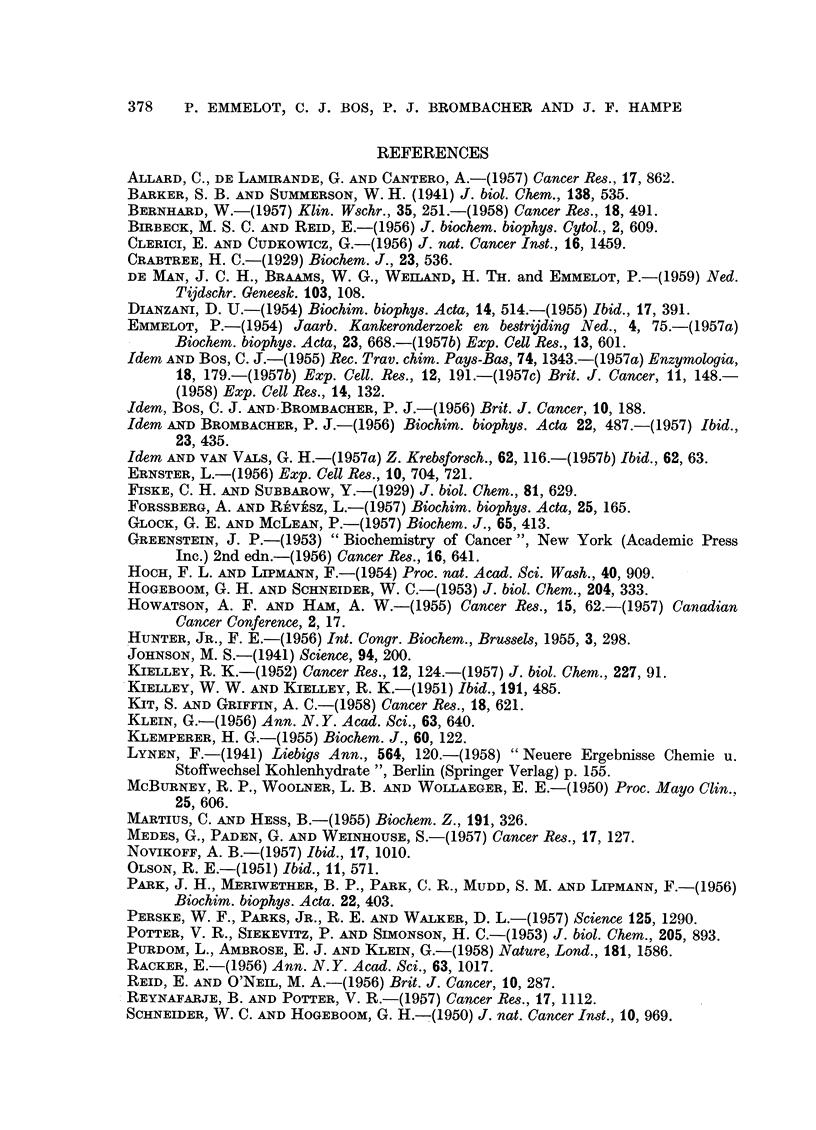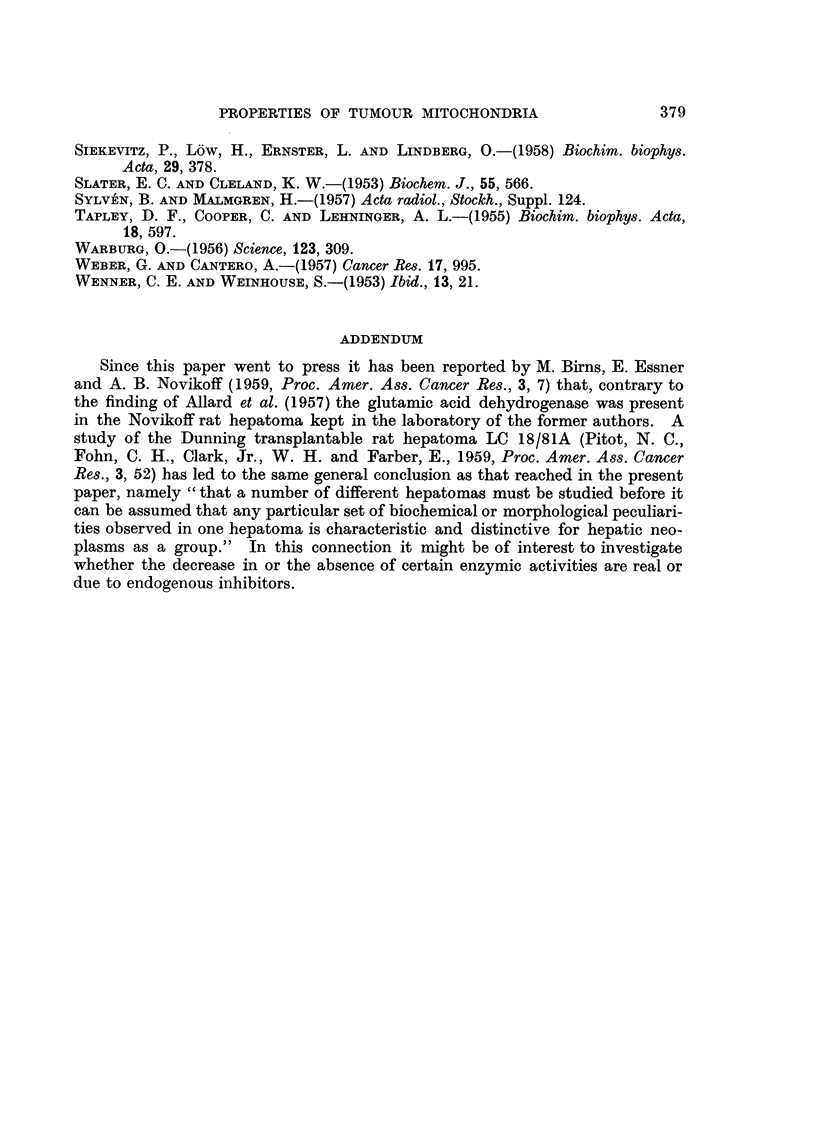# Studies on Isolated Tumour Mitochondria: Biochemical Properties of Mitochondria from Hepatomas with Special Reference to a Transplanted Rat Hepatoma of the Solid Type

**DOI:** 10.1038/bjc.1959.42

**Published:** 1959-06

**Authors:** P. Emmelot, C. J. Bos, P. J. Brombacher, J. F. Hampe

## Abstract

**Images:**


### STUDIES ON ISOLATED TUMOUR MITOCHONDRIA: BIOCHEMICAL PROPERTIES OF MITOCHONDRIA FROM HEPATOMAS WITH SPECIAL REFERENCE TO A TRANSPLANTED RAT HEPATOMA OF THE SOLID TYPE

P. EMMELOT, C. J. BOS, P. J. BROMBACHER AND J. F. HAMPE

*From the Departments of Biochemistry and Pathological Anatomy, Antoni van Leeuwenhoek-Huis: The Netherlands Cancer Institute, Amsterdam, The Netherlands*

Received for publication January 8, 1959

A BASIC policy in the study of neoplastic growth is to compare the metabolic characteristics of a tumour with those of the homologous normal tissue. In the long run this may lead to a better understanding of the neoplastic diseases and furnish some rational clue(s) for a chemotherapy of cancer.

The rat liver is a suitable tissue for study because of its relatively uniform composition, the ease of obtaining a sufficient quantity of tissue and the possibility of inducing experimental hepatomas with chemical agents. In the experiments to be reported in the present paper, mitochondria were isolated from a transplanted rat hepatoma and studied in respect to a number of important enzymic reactions. The original tumour (case No. BY 252) arose several years ago in a rat of our inbred colony as a result of feeding DAB*. The following favourable circumstances allow that a strict comparison can be made between the enzymic activities of the hepatoma and normal liver mitochondria. The intraperitoneally transplanted BY 252 rat hepatoma is composed of a uniform population of undifferentiated liver tumour cells classified as a carcinoma solidum. Since fibrous elements are virtually absent, the tumour shows the same softness as normal rat liver and homogenization of the two types of tissue can thus be performed in exactly the same way.

The levels of the free and DNP-activated ATPases, oxidative phosphorylation in the absence and presence of thyroxine and fatty acid oxidation of the hepatoma particles have been studied. The results of these experiments clearly showed that the hepatoma mitochondria were more labile *in vitro* than normal rat liver mitochondria.

Another transplanted rat hepatoma which has become a favourite test object in recent years is the Novikoff hepatoma (Novikoff, 1957). The latter tumour and the BY 252 hepatoma show approximately the same rate of growth and a similar histological structure; both have been induced by DAB. According to the Greenstein concept, undifferentiated tumours, as a class, tend to resemble each other metabolically more than they do their tissue of origin (Greenstein, 1953, 1956). The pertinent data on which this concept is based, apply not only to metabolic functions performed by organo-typical enzyme systems—such as those mediating the formation of urea in the liver—but also to enzymes which are

* Abbreviations are used as follows: DAB = *p*-dimethylaminoazobenzene, AAT = *o*-aminoazotoluene, ATP = adenosine triphosphate, DNP = 2,4 : dinitrophenol, DPN = diphosphopyridine nucleotide, NAA = nicotinamide, EDTA = ethylenediamine tetra-acetate, $\alpha$-OC = $\alpha$-oxycaproate.

among the basic functional equipment of practically every mammalian cell. The mitochondrial glutamic dehydrogenase and ATPase are considered to belong to the latter class of enzymes. Accordingly, it may be expected that the mitochondrial activities of the BY 252 hepatoma would bear more resemblance to those of the Novikoff hepatoma than to those of normal rat liver. The statement that " the glutamic dehydrogenase is the first enzyme known to be lacking in mitochondria of liver tumours " (Allard, de Lamirande and Cantero, 1957), based on the finding that no trace of the enzyme could be detected in isolated mitochondria or homogenates of the Novikoff hepatoma, might thus be warranted and have some genuine significance for the problem of neoplastic liver growth if a similar situation should exist in the case of the BY 252 hepatoma. The same consideration applies to the finding (Novikoff, 1957) that addition of DNP to homogenates of the Novikoff hepatoma does not lead to an enhanced dephosphorylation of ATP, which suggests that the tumour mitochondrial ATPases, in contrast to the corresponding enzymes in liver mitochondria, are in a non-latent state.

It will be shown, however, that a marked difference between the Novikoff and the BY 252 hepatomas does exist in that the particles of the latter tumour contain a high glutamic dehydrogenase activity and also latent ATPase activity. These results lead one to regard the two tumours as separate biological entities, each with its own distinct metabolic background. Accordingly, only the study of a number of tumours derived from the same tissue might reveal a common characteristic which distinguishes the particular tumour type from its homologous normal tissue.

Substantiation for a possible generalization of the concept that hepatoma mitochondria, as a class, are more labile than liver mitochondria has therefore been sought in the study of a number of different hepatomas, both primary and transplanted. For reasons of comparison a study of the mitochondria from a mammary ascites carcinoma of the mouse and from a sarcoma has been included.

### MATERIALS AND METHODS

*Tumours.*—Liver tumours were induced over a period of 4 years by feeding DAB to approximately 250 female rats of the inbred strain R-Amsterdam which were kept on a diet of polished rice. Hepatomas of the mouse were obtained by administration of AAT to 125 CBA females fed *ad libitum* with the standard laboratory diet (Emmelot and van Vals, 1957*a*). Administration of the azo dyes was started when the animals were 1–2 months (rats) and 2–3 months (mice) of age. The rats received DAB (10 mg. in 0·2 ml. rape-oil) each day except at week-ends and the mice received AAT (1 mg.) once weekly at the start and increasing to twice, thrice weekly etc. until after 5 months AAT was administered each day except at week-ends. Rat hepatomas appeared after 4–12 months and mice hepatomas after 11–12 months. The administration was continued until the tumours became palpable. For certain experiments the animals were sacrificed before the tumours became palpable. In some experiments mouse liver together with small hepatoma nodules was used.

After cervical dislocation under ether anesthesia, the liver with adhering tumour(s) was quickly removed and hepatoma tissue was completely separated from the remaining liver. Representative parts of the tissues were fixed, stained and subjected to microscopic examination. A detailed study was made of the histological structure of the rat tumours and livers. The expression " hepatoma " is used in the present investigation to denote malignant liver growth. The rat tumours were classified as solid (hepatocellular), adeno (cholangiocellular) or mixed types in a descriptive sense. Only the experiments with solid hepatomas are included in the present report. In addition a more differentiated solid type of tumour, *i.e.* the trabecular hepatoma, resembling liver tissue, was recognized. The histological structures, illustrating the case report of the BY 252 rat hepatoma (Fig. 1–6), are representative for the liver tumours observed in our rats.

*The BY 252 rat hepatoma.*—A hepatoma which arose in January 1956 in one of our rats (BY 252), following the ingestion of DAB during 5 months, was successfully transplanted by subcutaneous grafting. The primary tumour was of the mixed adeno-solid type (Fig. 1 and 2). In the slice prepared from the first transplant, adenohepatoma was found (Fig. 3). Until the 28th transplant generation the tumour was not subjected to further microscopic examination. The latter transplant was composed of solid hepatoma (Fig. 4). Subcutaneous transplantation led to a marked necrosis. The tumour was, therefore, subsequently transferred by the intraperitoneal route. At the moment the tumour is transplanted each ninth day. During this period the hepatoma grows rapidly as largely non-necrotic soft masses throughout the mesentery and peritoneum. When the tumour was allowed to grow longer, marked necrosis was found and the animals suddenly died (14 days). Some ascites formation was always observed ; it was possible to transplant the tumour by using a small amount of ascites fluid—tumour masses always resulted. All transplants took in 100 per cent of the cases.

The intraperitoneally transplanted BY 252 rat hepatoma consists of undifferentiated hepatoma cells (Fig. 6) with a small rim of a more differentiated cell type which infiltrates the peritoneal fat (Fig. 5). Fibrous elements are virtually absent. The hepatoma is classified as a carcinoma solidum of the spindle cell type. Unless stated otherwise, only intraperitoneal transplants have been used in the present experiments.

Apart from the above tumours, another type of growth—resembling liver in colour and softness—was encountered in about 20 of the rats on the DAB diet. The fresh weight of these tissues varied from 5–40 grammes. Both histological and biochemical criteria allow the conclusion that these growths are of a non-malignant character. A detailed description of the histology (Fig. 7–12) of one case (rat BY 235, fed with DAB during 14 months) is given.

*Rat BY 235*

*Macroscopy.*—The right and middle liver lobes are without gross pathology. The left lobe is changed into a spherical " tumour " of 40 g. wet weight (the rest of the liver weighing about 5 g.). The cut surface has a speckled yellowish gray brown appearance. The " tumour " is partly surrounded by a thin layer of atrophic fatty liver tissue.

*Microscopy.*—As usual in these experiments, cellular and nuclear pleomorphism with fatty acid infiltration in right and middle lobes. No cirrhosis. Scattered small nodules of hyperplasia which may be basophilic (Fig. 11) or eosinophilic (Fig. 12). There are only occasional small foci of haemopoiesis. *Left lobe* : the " tumour " consists of a mixture of fatty cells and hyperplastic cells of basophilic type

(Fig. 7, 8 and 10). The picture is principally the same as in the small nodules in the right lobe. The hyperplastic cells are more pleomorphic than in the (altered) surrounding liver tissue and in the (altered) right lobe. It is impossible to state whether the fatty cells in the " tumour " are original liver cells or fatty infiltrated hyperplastic cells. In the " tumour " many foci of haemopoiesis (Fig. 9) are found ; far more than in the rest of the liver.

In ordinary malignant hepatomas haemopoiesis was found only as long as original liver tissue was included in the tumour. The whole swelling of the left lobe is histologically interpreted as to be of a non-malignant, not definitely tumourous, but hyperplastic nature. The name " hyperplastoma " may serve to distinguish this type of growth from hepatoma (the expression " hepatoma of low malignancy " is sometimes also used in the literature). These large, local hyperplastomas may be principally of the same nature as the small foci of hyperplasia that one finds so often scattered throughout the pre-neoplastic rat liver. No relation appeared to exist between these small foci and cirrhosis, although they are often combined. They should not be considered to be identical with the nodules in annular cirrhosis. In our opinion there exists a relation between these foci of hyperplasia and true (malignant) hepatoma, since isolated areas of small amounts of hepatoma cells could be observed in the hyperplastomas and hyperplastic foci. We think it possible that this form of hyperplastoma has its counterpart in the isolated cirrhotic hyperplastic " tumours " of the liver as seen in man (McBurney, Woolner and Wollaeger, 1950).

The transplanted mouse tumours (hepatomas and sarcoma UV 256), used in the present investigation, were well-established specimens induced and mantained in our laboratory for more than five years. The ascites tumour studied was the S3A mammary carcinoma originally obtained from Professor G. Klein (Stockholm).

*Isolation of mitochondria.*—The excised tissues were collected in small beakers placed in crushed ice and immediately cut into small pieces and homogenized at 0° C. in an all-glass Potter-Elvejhem type of apparatus with a loose-fitting pestle. Ascites cells were harvested from $F_1(B \times C_3H)$ mice inoculated 7–9 days before. The ascites fluid was removed and the cells were freed from erythrocytes by one suspension in physiological saline followed by low-speed centrifugation at 0° C. The cells were suspended in twice distilled water for 10–15 minutes and immediately homogenized after addition of sucrose and EDTA to give a final concentration of 0·25 and 0·001 M, respectively. The homogenization was carried out by using a rather tight-fitting pestle ; in some experiments the ascites cells were collected and immediately disrupted by sonic oscillation during 105 seconds (9 kc). One part of tissue and 9 parts of medium were used in the case of liver and liver tumours and 4–5 parts of medium with other tumours. In most experiments 0·25 M sucrose containing 0·001 M EDTA, pH 7·4, has been used as isolation medium for the mitochondria. As indicated in the text 0·25 M sucrose alone, or 0·25 M sucrose containing 0·01 M EDTA and 0·001 M NAA, has been used in certain experiments. In still other cases 0·23 M raffinose hydrate, 0·001 M EDTA and 0·085 per cent heparine in 6 per cent dextran served as medium (Birbeck and Reid, 1956). The dextran (MW 150·000) originally used was a gift from Glaxo Laboratories ; for later experiments dextran of MW 75·000 was purchased from Poviet Cy., Amsterdam, and included in the medium in a final concentration of 3 per cent.

Homogenization of the cells and finely-cut tissues was carried out by gently passing the tube, 6 times along the rotating pestle (1400 r.p.m.), which had to be carefully centered, avoiding too much manual pressure. The homogenization lasted for about 25–35 seconds ; any tissue left intact after this treatment was discarded.

The homogenates were centrifuged for 10 minutes at 700 × g and 0° C. Only the middle part of the tube content was used for the subsequent isolation of the mitochondria ; the surface layer, which mainly consisted of fat droplets, was carefully removed and a rather ample layer above the nuclear pellet was discarded. This procedure appeared to be important in order to obtain tumour mitochondria with a relatively low ATPase activity. The nuclear pellet was not washed. Mitochondria were spun down at 5000 × g during 10 minutes (the low gravitational field was chosen in order to minimize the risk of microsomal contamination), the " fluffy layer was removed and the pellet was resuspended and washed once (ATPase assay) or twice (respiration study). The fresh mitochondria were always immediately used. In a number of experiments the particles were disrupted either by freeze-thawing (— 15° C. → + 5° C ; 3 or 6 times) or by sonic oscillation with 9 kc at 50 Watt in the Raytheon oscillator during 10 minutes, unless otherwise stated.

*Measurements.*—For the ATPase assay 0·1 ml. mitochondrial suspension and 0·1 ml. 0·1 M ATP was added to 1·6 ml. containing 0·1 M KCl, 0·05 M TRIS and 0·005 M $MgSO_4$, pH 7·2; DNP present in a final concentration of $10^{-4}$ M. Incubation unless otherwise stated at 27° C. Reaction stopped by adding 0·1 ml. 50 per cent trichloroacetic acid. Inorganic phosphate measured according to Fiske and Subbarow (1929).

The experiments in which the oxidative properties of the primary rat and mouse hepatoma mitochondria were studied (Tables X and XI) have been conducted as described earlier (Emmelot and Bos, 1955, 1957*a*, at pH 7·4 in phosphate buffer). The other experiments (Tables VIII, XI, XII and XIII) have been carried out in histidine buffer pH 7·0 (Emmelot, 1957*a*). The experiments with thyroxine were always performed at pH 7·4 either in phosphate or in histidine buffer. 0·1 ml. of DL-thyroxine (obtained from Hofmann and la Roche) in 0·004 M NaOH was added to the respirometer flask to give a final concentration of $7{\cdot}5 \times 10^{-5}$ M. Total fluid volume was always 1·6 ml. Hexanoate and octanoate 2 $\mu$moles, pyruvate 24 $\mu$moles, glutamate 56 $\mu$moles, $\beta$-hydroxybutyrate 12 $\mu$moles. The $O_2$-consumptions have always been corrected for blanks obtained in the absence of the particular substrates.

It should be noted that in those experiments of Table X and Table XI in which $\alpha$-oxycaproate (0·003 M) was added as a primer of fatty acid oxidation (Emmelot and Bos, 1955), octanoate is completely converted to acetoacetate when 134 $\mu$l of $O_2$ are consumed. In the presence of L-malate (0·0003 M), however, oxidation via the citric acid cycle becomes possible. In a number of experiments DL-$\beta$-hydroxybutyrate was incubated in the absence of a primer since the oxidation of the D(—)isomer to acetoacetate, in contrast to that of the L(+)isomer, is independent of ATP and coenzyme A. 1 Mg. hexokinase (grade III, Sigma) containing 153 K.M. units at 25° C was added as indicated. The serum albumin used was of human origin and prepared in the Central Laboratory of the Netherlands Red Cross (Amsterdam). It was thoroughly dialyzed before use to remove oxidizable substrates.

### RESULTS

### *ATP-Dephosphorylation*

Freshly-prepared intact mitochondria of rat liver contain a so-called latent adenosine triphosphatase. ATPase activity can be manifested in these particles either by 2 : 4-dinitrophenol (DNP-activated ATPase) or by physical and chemical agents which cause damage to the integrated structure of the mitochondria ($Mg^{2+}$-activated ATPase) (Kielley and Kielley, 1951 ; Potter, Siekevitz and Simonson, 1953 ; Siekevitz, Löw, Ernster and Lindberg, 1958). The DNP-activated ATPase is generally considered (Hunter, 1956) to represent a reversal and diversion of the reactions which are responsible for the synthesis of ATP during the process of oxidative phosphorylation, whereas the $Mg^{2+}$-activated ATPase probably represents some mal-function resulting from a disorganization of the otherwise integrated enzymic machinery of the latter process. It has been shown that progressive ageing of isolated liver mitochondria leads to a labilization of the integrated functions as shown by the loss of DPN, a gradual decrease in the efficiency of oxidative phosphorylation and an increase in free ($Mg^{2+}$-activated) ATPase. Concomitantly, the DNP-activated ATPase decreases so that eventually a situation may be reached in which DNP no longer activates the ATPase in the presence of $Mg^{2+}$ (Potter *et al.*, 1953 ; Siekevitz *et al.*, 1958). Hence the presence of free ATPase activity and the level of the DNP-activated ATPase relative to the former activity, may provide some information about the degree of intactness of the biochemical features of isolated mitochondria.

*The BY 252 hepatoma, $S_3A$ ascites carcinoma and UV 256 sarcoma*

Table I illustrates the dephosphorylation of ATP by fresh mitochondria isolated from the BY 252 rat hepatoma in 0·25 M sucrose containing 0·001 M EDTA and incubated at 27° and 37° C in the absence and presence of $Mg^{2+}$ and/or DNP ($10^{-4}$ M). Dephosphorylation is recorded as the micrograms phosphor liberated from ATP. On account of the non-proportionality of enzyme activity to enzyme concentration (Potter *et al.*, 1953 ; Emmelot, Bos and Brombacher, 1956) no attempt has been made to express the present data on a uniform weight basis. Since some variation was observed with individual preparations each experimental condition has been illustrated by a number of results.

ATP-dephosphorylation in the absence of $Mg^{2+}$ and DNP varied from almost zero to moderate values (experiments 2*c*, 3*e*, and 4*e*, respectively). In the presence of DNP and the absence of $Mg^{2+}$ (experiments 2*d* and 4*f*), and *vice versa* (experiments 2*a* and 4*a*), enhanced activity was noted. The fresh hepatoma mitochondria thus contained a $Mg^{2+}$- and a DNP-activated ATPase at 27° C. The $Mg^{2+}$-activated ATPase of the fresh tumour particles was more active and the DNP-activated enzyme less active than the corresponding enzymes in the mitochondria of normal liver of rats of the same inbred strain as that from which the hepatoma was derived (Table II).

The ATP-dephosphorylation by the hepatoma mitochondria at 27° C in the presence of DNP and $Mg^{2+}$ was always higher than that in the presence of $Mg^{2+}$ alone (experiments 1*b*, 2*b*, 3*b*, 4*b*, 5*b*). Thus, in the presence of free ATPases the DNP-activated ATPase could be manifested. However, it is evident that the effect of DNP is smaller at higher levels of the free ($Mg^{2+}$-activated) ATPase. The latter is more clearly illustrated by the results of similar experiments carried out at 37° C.

The $Mg^{2+}$-activated ATPase of the hepatoma mitochondria was always more active at 37° C (experiments 3*c*, 4*c* and 5*b*) than at 27° C. Apart from the

TABLE I.—*Effect of DNP and/or $Mg^{2+}$ on ATP-dephosphorylation by fresh mitochondria from the BY 252 rat hepatoma at 27° and 37° C.*

Isolation mitochondria in 0·25 M sucrose containing 0·001 M EDTA.

| Experiment No. | Amount of mitochondria (mg. N/flask) | Temperature of incubation (°C.) | $Mg^{2+}$ | DNP | μg. phosphor released after minutes 5 | 10 | 20 |
|---|---|---|---|---|---|---|---|
| 1 *a* | 0·04 | 27 | + | — | 8 | 12 | 16 |
| *b* | | 27 | + | + | 12 | 40 | 65 |
| 2 *a* | 0·04 | 27 | + | — | 2 | 14 | 34 |
| *b* | | 27 | + | + | 18 | 27 | 48 |
| *c* | | 27 | — | — | 0 | 2 | 6 |
| *d* | | 27 | — | + | 9 | 12 | 16 |
| 3 *a* | 0·07 | 27 | + | — | 6 | 17 | 34 |
| *b* | | 27 | + | + | 30 | 42 | 51 |
| *c* | | 37 | + | — | 11 | 31 | 58 |
| *d* | | 37 | + | + | 30 | 50 | 68 |
| *e* | | 37 | — | — | 0 | 11 | 20 |
| 4 *a* | 0·07 | 27 | + | — | 15 | 25 | 63 |
| *b* | | 27 | + | + | 28 | 51 | 92 |
| *c* | | 37 | + | — | 22 | 46 | 96 |
| *d* | | 37 | + | + | 33 | 52 | 94 |
| *e* | | 27 | — | — | 7 | 17 | 25 |
| *f* | | 27 | — | + | 32 | 42 | 67 |
| 5 *a* | 0·05 | 27 | + | — | 8 | 25 | 36 |
| *b* | | 27 | + | + | 33 | 62 | 70 |
| *c* | | 37 | + | — | 26 | 49 | 75 |
| *d* | | 37 | + | + | 36 | 58 | 80 |

TABLE II.—*Effect of DNP on ATP-dephosphorylation by fresh rat liver mitochondria at 27° and 37° C.*

Isolation of mitochondria in 0·25 M sucrose containing 0·001 M EDTA.

| Amount of mitochondria (mg. N/flask) | Temperature of incubation (°C.) | $Mg^{2+}$ | DNP | μg. phosphor released after minutes 5 | 10 | 20 |
|---|---|---|---|---|---|---|
| 0·04 | 27 | — | — | 0 | 0 | 2 |
| | 27 | — | + | 25 | 61 | 95 |
| | 27 | + | — | 0 | 5 | 15 |
| | 27 | + | + | 40 | 65 | 118 |
| | 37 | + | — | 8 | 14 | 31 |
| | 37 | + | + | 50 | 105 | 156 |
| 0·06 | 27 | + | — | 0 | 0 | 8 |
| | 27 | + | + | 60 | 89 | 123 |
| 0·05 | 37 | + | — | 18 | 28 | 36 |
| | 37 | + | + | 67 | 102 | 155 |

effect of the temperature on the reaction rate, the higher activity might also have been due to an increased labilization of the particles, which developed the $Mg^{2+}$-activated ATPase more or less completely and thus depressed the reaction sequence of the DNP-activated ATPase. The absence of any marked effect of DNP on the ATP dephosphorylation in the presence of $Mg^{2+}$ at 37° C observed in two experiments (experiments 4*d* and 5*d*) during the whole period of incubation shows that the latter situation may indeed be realized. In another experiment (3*c*) the particles were somewhat more resistant to the higher temperature so that the $Mg^{2+}$-activated ATPase did not develop completely and DNP (experiment 3*d*) still showed some effect.

As illustrated in Table III mitochondria prepared from the S3A ascites mammary carcinoma behaved similarly. A marked effect of DNP on ATP-dephosphorylation in homogenates of ascites cells was also observed at 27° and 37° C. Microscopic analysis has shown that in these homogenates approximately 10 per cent intact cells were present. The effect of DNP on the ATP-dephosphorylation of the homogenates was not due to a stimulation of the ATPase of the remaining intact cells since in parallel experiments it was found that DNP exerted a negligible effect on the ATP-dephosphorylation of freshly harvested ascites cells at 27 and 37° C. The ATPase of sarcoma mitochondria (tumour UV 256 of the mouse) may also be activated by DNP at 27° C. In the two experiments illustrated in Table III the latter effect was very evident ; in a number of other experiments a less distinct effect was observed.

TABLE III.—*Effect of DNP on ATP-dephosphorylation by mitochondria and homogenates prepared from the S3A ascites carcinoma and by mitochondria from the UV 256 sarcoma.*

Isolation mitochondria in 0·25 M sucrose containing 0·001 M EDTA. Incubation in the presence of $Mg^{2+}$.

| Source of enzyme | Temperature of incubation (°C.) | DNP | $\mu$g. phosphor released after minutes 5 | 10 | 20 |
|---|---|---|---|---|---|
| S3A ascites mitochondria | 27 | — | 8 | 15 | 30 |
| (0·03 mg. N/flask) | 27 | + | 20 | 35 | 61 |
| | 37 | — | 14 | 36 | 90 |
| | 37 | + | 42 | 62 | 96 |
| S3A ascites homogenate | 27 | — | 10 | 16 | 31 |
| (4·7 mg. dry weight/flask) | 27 | + | 30 | 37 | 53 |
| Ditto | 37 | — | 10 | 19 | 45 |
| (4·0 mg. dry weight/flask) | 37 | + | 55 | 80 | 85 |
| UV 256-sarcoma mitochondria | 27 | — | .. | 21 | 52 |
| (0·12 mg. N/flask) | 27 | + | .. | 80 | 148 |
| (0·10 mg. N/flask) | 27 | — | .. | 35 | 58 |
| | 27 | + | .. | 64 | 103 |

In the case of normal rat liver mitochondria the higher incubation temperature (37° C) evoked also the $Mg^{2+}$-activated ATPase but the level of this activity at 37° C was, in contrast to the situation in the hepatoma mitochondria, only a small fraction of that reached when DNP was present in addition (Table II).

The ATP-dephosphorylation by disintegrated hepatoma and liver mitochondria is illustrated in Table IV. The particles were either freeze-thawed or sonicated to ensure complete structural disorganization and, thus, to uncover the potential ATPase content. ATP-dephosphorylation by the disrupted liver and hepatoma particles in the presence of $Mg^{2+}$ was similar to that observed with untreated particles in the presence of DNP and $Mg^{2+}$. Addition of DNP to the disrupted mitochondria had little effect in the case of liver and no effect at all in the case of hepatoma ($Mg^{2+}$ present).

TABLE IV.—*Effect of DNP on ATP-dephosphorylation by fresh and disrupted hepatoma and liver mitochondria at 27° C*

Mitochondria isolated in 0·25 M sucrose containing 0·001 M EDTA. Incubation in the presence of $Mg^{2+}$.

| Mitochondria from (mg. N/flask) | Condition | DNP | μg. phosphor released after minutes 5 | 10 |
|---|---|---|---|---|
| BY 252 rat hepatoma (0·07) | Fresh | – | 9 | 15 |
| | | + | 18 | 40 |
| | Sonicated | – | 20 | 33 |
| | | + | 18 | 40 |
| | Freeze-thawed | – | 20 | 35 |
| | | + | 24 | 35 |
| (0·05) | Fresh | – | 4 | 10 |
| | | + | 12 | 26 |
| | Sonicated | – | 11 | 24 |
| | | + | 13 | 25 |
| Rat liver (0·07) | Fresh | – | 0 | 7 |
| | | + | 59 | 97 |
| | Sonicated | – | 55 | 99 |
| | | + | 63 | 113 |

The data presented in Tables I to IV allow the conclusion that freshly prepared mitochondria of the BY 252 rat hepatoma are more labile than rat liver mitochondria and especially so at a temperature of 37° C. The enhanced lability of the hepatoma particles leads to the spontaneous development of the $Mg^{2+}$-activated ATPase and to a decrease in activity of the DNP-activated enzyme. However, as long as the $Mg^{2+}$-activated enzyme is not completely manifest, a distinct effect of DNP can be observed. It appears further that the DNP-activated ATPase of the hepatoma particles, both in the absence and in the presence of $Mg^{2+}$, is smaller than that of liver mitochondria. This applies also to the potential ATPase-content, as manifested in disrupted mitochondria by $Mg^{2+}$.

The data of Table I show that separate mitochondrial preparations of the BY 252 rat hepatoma may vary somewhat in the degree of lability, This variability was also observed in the efficiency of oxidative phosphorylation and fatty acid oxidation of the particles (compare below). This phenomenon is probably connected with the physiological state of the tumour since "young" tissue (less than 9 days after transplantation) gave the best results.

*Primary rat hepatomas, rat liver hyperplastomas, primary and transplanted mouse hepatomas.*—Mitochondria from primary DAB-induced hepatomas of the solid type and from certain other tumours (Emmelot, Bos and Brombacher,

1956 ; Emmelot and Bos, 1957*c* ; Potter *et al.*, 1953) have earlier been found to possess an appreciable free ATPase activity which, except in the case of spontaneous mouse hepatomas, could not be stimulated by DNP. A lack of response of the ATP-dephosphorylation to DNP has been observed in homogenates of primary rat hepatomas and other tumours (Novikoff, 1957). Free ATPase activity in particles of primary rat hepatomas (15 minutes at 12·000 × g., " fluffy layer " not removed) has also been noted by other investigators (Reid and O'Neil, 1956). The latter results are, however, difficult to interpret since microsomal material (which in tumours may possess appreciable ATPase activity) of the " fluffy layer " might have contributed to the observed activities.

After carrying out the isolation as described under Materials and Methods we have been able in the last two years to prepare mitochondria from primary rat hepatomas (0·25 M sucrose containing 0·00 1M EDTA) which show an enhanced ATP-dephosphorylation on addition of DNP at 27° C. Some of these results, including those obtained with mitochondria from mouse hepatomas, are listed in Table V. In these experiments the level of the free ATPase and the effect of DNP was of the same order as that observed with the BY 252 hepatoma mito-

TABLE V.—*Effect of DNP on ATP-dephosphorylation by fresh mitochondria from primary rat and mouse hepatomas, transplanted mouse hepatomas and rat hyperplastomas*

Mitochondria isolated in 0·25 M sucrose containing 0·001 M EDTA. Incubation in the presence of $Mg^{2+}$.

| Mitochondria from | Amount of mitochondria (mg. N/flask) | DNP | μg. phosphor released after minutes 5 | 10 | 20 |
|---|---|---|---|---|---|
| Primary rat hepatomas, solid type | 0·07 | − | 8 | 18 | 33 |
| | | + | 9 | 20 | 64 |
| | 0·05 | − | 7 | 13 | 23 |
| | | + | 14 | 27 | 49 |
| | 0·06† | − | 6 | 12 | 35 |
| | | + | 26 | 42 | 55 |
| | 0·06 | − | 6 | 10 | 35 |
| | | + | 12 | 25 | 45 |
| Primary mouse hepatomas | 0·04 | − | 0 | 5 | 14 |
| | | + | 5 | 26 | 52 |
| | | +* | 30 | 42 | 58 |
| | 0·30 | − | 46 | 90 | 142 |
| | 0·27 | − | 26 | 65 | 87 |
| Transplanted mouse hepatomas : | | | | | |
| T 28012 | 0·04 | − | 6 | 13 | 25 |
| | | + | 12 | 26 | 51 |
| T 26473 | 0·04 | − | 8 | 17 | 34 |
| | | + | 16 | 35 | 59 |
| | 0·25 | − | 41 | 68 | 114 |
| Hyperplastoma, rat | 0·05 | − | 5 | 8 | 15 |
| | | + | 11 | 24 | 45 |
| Hyperplastoma/solid hepatoma | 0·06 | − | 3 | 13 | 32 |
| | | + | 37 | 67 | 102 |
| (With some cirrhotic liver rests) | 0·03 | − | 0 | 6 | 18 |
| | | + | 25 | 36 | 62 |

* γ-globulin added (compare Emmelot and Bos, 1957c).
† BY 400, compare for liver Table VI.

chondria. Sometimes the effect of DNP developed sluggishly (compare the first experiment of Table V). Disrupted mitochondria of primary rat hepatomas did not show a higher ATP-dephosphorylation ($Mg^{2+}$ present) than that observed with fresh particles in the presence of DNP and $Mg^{2+}$.

The mitochondria of some primary AAT-induced mouse hepatomas and of all " hyperplastomas ", found in rat livers as a result of DAB feeding, showed a more " normal " behaviour in the presence of DNP (Table V). The oxidative properties of the hyperplastoma mitochondria have also been found to resemble more those of normal liver mitochondria than of malignant hepatoma mitochondria (Table X). The non-tumourous character of the hyperplastomas is further illustrated by the following biochemical criteria. The glucose-6-phosphate dehydrogenase, the reduced triphosphopyridine nucleotide diaphorase and -cytochrome *c* reductase contents of the combined microsomal soluble fractions of the hyperplastomas have in four experiments been found to be similar to those of liver of control-rats fed with polished rice for prolonged periods and of liver from which hepatomas (13 experiments) had been removed. The levels of these three enzymes amounted to, respectively, 120, 50 and 50 per cent of those of livers of rats fed the standard diet. In solid, adeno or mixed hepatomas (10 experiments) the glucose-6-phosphate dehydrogenase had increased to 200–300 per cent and the diaphorase and cytochrome *c* reductase dropped to 10–20 per cent of the normal level. These latter data may be compared with those reported for the Novikoff hepatoma, which show that the glucose-6-phosphate dehydrogenase is increased for 500 per cent (Weber and Cantero, 1957) whereas the TPNH-cytochrome *c* reductase was absent (Reynafarje and Potter, 1957).

*Pre-neoplastic rat liver.*—Since the DAB-fed rats had been placed on a protein- and vitamin-deficient diet, it was investigated next whether this might have had any effect on the level of the mitochondrial ATPases. Liver mitochondria of rats receiving the standard laboratory diet and of rats fed polished rice showed the same low ATPase activity and the effect of DNP was also similar in the two cases, both at pH 7·2 and 8·5 (Table VI). At pH 8·5, the free ATPase activity was higher and the DNP-activated ATPase lower than the corresponding activities at pH 7·2. ATP-dephosphorylation by fresh liver mitochondria in the presence of $Mg^{2+}$ and DNP at pH 7·2 was equal to that obtained with sonically disrupted particles in the presence of $Mg^{2+}$. However, at pH 8·5 the ATP-dephosphorylation under the latter conditions was distinctly higher than that of fresh particles incubated in the presence of $Mg^{2+}$ and DNP.

Mitochondria from liver tissue dissected free from adhering hepatomas or from livers in which the hepatomas had not yet appeared behaved more or less like normal liver mitochondria; the free ATPase was sometimes enhanced (compare Clerici and Cudkowicz, 1956). These livers showed one or more of the following pathological changes: cirrhosis, fatty infiltration, hyperplastic and regenerative foci, cell and nuclear polymorphy and cholangiofibrosis. In some mitochondrial preparations of livers in which the latter changes were very marked, DNP was less active (last experiments of Table VI and Fig. 13).

*Abolishment of Pasteur-effect in hepatoma slices by DNP.*—An effect of DNP on mitochondrial processes could also be shown with intact tumour cells (Emmelot and Bos, unpublished). The anaerobic glycolysis of the tumours (at 37° C, Table VII) was higher than the aerobic glycolysis, but in the presence of DNP the aerobic lactate production was equal to the anaerobic one. In view of the inter-

TABLE VI.—*Effect of DNP on ATP-dephosphorylation by fresh and disrupted liver mitochondria of rats fed the standard laboratory diet, polished rice and polished rice plus DAB*

Incubation during 10 minutes at 27° C at pH 7·2 and 8·5, in the presence of $Mg^{2+}$. Isolation of mitochondria in 0·25 M sucrose containing $10^{-3}$ M EDTA (*s* + *v*) or in raffinose-dextran-heparine-EDTA (*r* + *d*).

The rats received polished rice during 6–7 months and polished rice plus DAB during 5–7 months (BY 387 and BY 400 : 7 months ; BY 384 A : 6 months ; BY 436 : 5 months).

| Liver Mitochondria from | Amount of mitochondria (mg. N/flask) | pH | μmoles phosphate released by mitochondria: Fresh − DNP | Fresh + DNP | Sonicated − DNP | Sonicated + DNP |
|---|---|---|---|---|---|---|
| Rat fed standard (s + v) | 0·14 | 7·2 | 0·3 | 3·8 | 3·8 | 4·6 |
| | | 8·5 | 1·0 | 3·3 | 4·6 | 5·6 |
| Rat fed polished rice (s + v) | 0·10 | 7·2 | 0·4 | 3·3 | 3·9 | 4·3 |
| | | 8·5 | 0·9 | 2·9 | 5·0 | 5·9 |
| Rat fed standard (r + d) | 0·09 | 7·2 | 0·2 | 3·0 | 2·9 | 3·7 |
| | | 8·5 | 0·6 | 1·6 | 4·0 | 5·5 |
| Rat fed polished rice (r + d) | 0·06 | 7·2 | 0·2 | 2·0 | 2·3 | 3·4 |
| | | 8·5 | 0·6 | 1·4 | 3·4 | 3·8 |
| Rats fed polished rice plus DAB, hepatomas removed : | | | | | | |
| BY 387, cirrhosis light degree (r + d) | 0·08 | 7·2 | 0·4 | 2·6 | .. | .. |
| | | 8·5 | 1·0 | 2·1 | .. | .. |
| BY 384 A, steatosis cell polymorphy, hyperplastic areas (r+d), | 0·06 | 7·2 | 0·2 | 1·6 | 2·3 | 2·7 |
| | | 8·5 | 0·5 | 1·2 | 3·6 | 3·6 |
| BY 400, severe steatosis (s + v) | 0·05 | 7·2 | 0·1 | 1·1 | .. | .. |
| | | | (0·2)* | (1·7)* | | |
| BY 436, whole liver affected by severe annular cirrhosis with cell polymorphy and marked cholangiofibrosis ; no hepatoma present† (s + v) | 0·03 | 7·2 | 0·4 | 0·9 | 1·2 | 1·4 |
| | | | (0·9)* | (1·8)* | (1·8)* | (2·1)* |

* After 20 minutes.

† Compare Fig. 13.

TABLE VII.—*The stimulatory effect of DNP on the aerobic glycolysis of rat hepatomas of the solid type*

Each flask contained 100 mg. of wet weight slices suspended in 1·6 ml. Krebs-Ringer bicarbonate buffer. Incubation was carried out during 45 and 60 minutes, respectively, at 37° C. Aerobic conditions : 95 per cent $O_2$ + 5 per cent $CO_2$ ; anaerobic conditions : 95 per cent $N_2$ + 5 per cent $CO_2$. $10^{-4}$ M DNP added as indicated. Lactic acid determined according to Barker and Summerson (1941).

| Tumour | Condition | DNP | Lactate produced (μmoles) |
|---|---|---|---|
| Primary hepatoma | Anaerobic | — | 10·1 |
| | Anaerobic | + | 9·2 |
| | Aerobic | — | 5·0 |
| | Aerobic | + | 8·9 |
| Transplanted BY 252 hepatoma | Anaerobic | — | 11·0 |
| | Anaerobic | + | 14·2 |
| | Aerobic | — | 8·4 |
| | Aerobic | + | 19·0 |

pretation of the Pasteur effect in terms of a competition between respiration and glycolysis for inorganic phosphate and adenine nucleotides (Johnson, 1941; Lynen 1941, 1958; Racker, 1956) it follows from the latter results that DNP uncoupled the oxidative phosphorylations in the respiratory chain of the tumour mitochondria *in situ*. Moreover, if the interpretation of the Pasteur effect is correct, its occurrence in slices of the hepatomas must mean that the ATPases of the mitochondria of intact tumour cells are at least in a partially, if not a completely, latent state at 37° C. The non-latency of the ATPase activity of the isolated BY 252 hepatoma mitochondria at 37° C is thus due to the *in vitro* conditions.

*Comment.*—The present results demonstrate that DNP may activate the ATPase of isolated tumour, including hepatoma, mitochondria. The reported absence of such an effect in homogenates of the Novikoff and primary rat hepatomas (Novikoff, 1957) stands in marked contrast to our results. Novikoff's suggestion that the lack of response to DNP might be a common property of undifferentiated tumours is therefore not warranted. The opposite is true, activation of the latent ATPase by DNP appears to be common property of mitochondria from both sarcomas and carcinomas, provided that the particles are isolated in a reasonably intact and pure condition. A drastic homogenization, poor isolation technique

---

### EXPLANATION OF PLATES

(All slides 6 mμ, fixation in Susa, stained with haematoxylin azophloxin.)

FIG. 1–6.—*The BY 252 rat hepatoma* (all magnifications × 550).

FIG. 1, 2.—Primary tumour (malignant hepatoma). Partly adenomatous mucous producing pattern with cellular and nuclear pleomorphism (Fig. 1); partly solid, trabecular, rather regular pattern resembling liver tissue (Fig. 2).

FIG. 3.—First subcutaneous transplant. The slide consists of adenocarcinoma (adeno-hepatoma) with marked pleomorphism and slight mucus production.

FIG. 4.—Subcutaneous transplant, 28th passage. Solid non-trabecular growing tumour with pleomorphism, marked necrosis and inflammation.

FIG. 5, 6.—Intraperitoneal transplant, 34th passage. The outer cells show more or less an epithelial pattern, infiltrating the peritoneal fat (Fig. 5). The rest (greatest part) of the tumour is composed of fusiform cells (sarcoid) with suggesting of interweaving bundles (Fig. 6). All further transplants were of similar structure (carcinoma solidum, spindle-cell type).

FIG. 7–12.—*Rat BY 235 liver* (5 g.) *and hyperplastoma* (40 g.).

FIG. 7.—" Hyperplastoma " in left lobe. Upper part of figure shows capsule of liver rest with fatty infiltration. The hyperplastoma contains " liver " cells (or newly formed hyperplastic cells) with fatty infiltration. The smaller, darker cells are of hyperplastic basophilic type. × 125.

FIG. 8, 9.—Borderline of same hyperplastoma as illustrated in Fig. 7. Rest of fatty liver is seen at top (Fig. 8, × 175). Small dark cells in hyperplastoma are foci of haemopoiesis, which are seen in detail in Fig. 9. × 500.

FIG. 10.—Detail of hyperplastoma. Trabecular arrangement of newly formed cells with patent capillaries lined by endothelium (without typical Kupfer-cells). × 500.

FIG. 11.—Basophilic focus of hyperplasia in right lobe. Fatty infiltration. No cirrhosis. × 30. Nuclear pleomorphism is evident at higher magnification.

FIG. 12.—Eosinophilic nodule of hyperplasia in fatty right lobe. × 30.

FIG. 13.—*Rat BY 436 liver.*

Nodular cirrhosis of entire liver (9 g.). Scale in centimetres. Microscopic: annular cirrhosis with pleomorphic " hyperplasia " of gall ducts (cholangiofibrosis).

1 2

3 4

5 6

Emmelot, Bos, Brombacher and Hampe.

7 8 9 10

11

12

13

Emmelot, Bos, Brombacher and Hampe.

and the use of mitochondria from " old " tumour transplants (necrosis) may, however, lead to negative results.

### *Oxidation and Phosphorylation*

*The BY 252 rat hepatoma : DPN-linked oxidations*

Tissue slices (150 mg. wet weight) of the 28th subcutaneous transplant series of the BY 252 rat hepatoma were incubated during 120 minutes with 0·25 $\mu$mol DL-glutamate-1-$C^{14}$ and approximately 25 per cent of the isotope was recovered in the respiratory carbon dioxide. Conclusive evidence for the presence of the glutamic dehydrogenase came from experiments conducted with the 33rd- and following intraperitoneal transplant generations, in which it was found that the mitochondria from these tumours, isolated in 0·25 M sucrose containing 0·001 M EDTA, were capable of oxidizing glutamate.

The oxidation of glutamate in the presence of DPN and NAA* was always high (Table VIII). A phosphorus to oxygen ratio of 1·0–1·8 in the absence and of 2·2–2·5 in the presence of the " high-energy " phosphate trapping system glucose-hexokinase was obtained in various experiments. The added hexokinase may thus compete successfully with the free ATPases of the particles for the ATP formed during oxidative phosphorylation. The variation in P : O ratio from 1·0–1·8 observed in the various experiments suggests that the free ATPase (or the degree of coupling) in some preparations was higher than in others. A similar conclusion was reached above.

When no DPN was added to the flasks the oxidation of glutamate by particles, isolated either in the presence or in the absence of 0·001 M NAA, but always incubated in the presence of NAA, was only 20–25 per cent of that recorded with DPN present.

*Pyruvate oxidation.*—The effect of DPN addition on the oxidation of pyruvate by mitochondria isolated from the BY 252 hepatoma in 0·25 M sucrose containing 0·001 M EDTA and 0·001 M NAA, was also studied (Table VIII). It appeared that the oxygen consumption in the absence of DPN was approximately 55 per cent of that found in the presence of DPN. It follows from these results that relatively more DPN was available to the enzymes oxidizing pyruvate than to those oxidizing glutamate. This may be connected with the site of the particular apoenzymes since the pyruvic dehydrogenase is known to be bound to the mitochondrial membranes whereas the glutamic dehydrogenase is located in the soluble part of the mitochondria (Hogeboom and Schneider, 1953). The poor $O_2$-consumption of the hepatoma particles in the absence of added DPN might have been due to the fact that the mitochondrial DPN-complement was already low *in situ* or that the DPN was lost from the mitochondria during their isolation and/or incubation. The loss could occur by the release of DPN (less intact membranes, less binding to proteins) or to the splitting of the coenzyme by an active DPNase. The latter was evidently not the case since the DPNase inhibitor NAA (added to the isolation or incubation medium) did not affect the rate of the oxidations. EDTA, on the other hand, which is known to protect against the loss of DPN from mitochondria (Slater and Cleland, 1953 ; Ernster, 1956 ; Emmelot and Bos,

* As a precaution NAA was added in all experiments to the respirometer flasks to prevent the enzymic splitting of DPN ; in a number of experiments NAA was omitted but no change in the oxygen consumption was observed, indicating that the DPNase was not very active.

TABLE VIII.—*Oxidation and phosphorylation by mitochondria from the transplanted BY 252 rat hepatoma*

Mitochondria isolated in 0·25 M sucrose containing $10^{-3}$ M EDTA, or $10^{-2}$ M EDTA (if marked with †), and $10^{-3}$ M NAA (if marked with *). Incubation as described (Emmelot, 1957*a*). NAA ($10^{-3}$ M) and DPN ($10^{-3}$ M) present in each flask which contained glutamate or pyruvate unless otherwise stated in parentheses. EDTA added to the incubation flasks as indicated in a final concentration of $5 \times 10^{-3}$ M EDTA. Hex-G stands for hexokinase and glucose. Pyruvate oxidation in the presence of L-malate (0·0003 M).

| Amount of mitochondria (mg. N/flask) | Time of incubation (min.) | Substrate | Additions | ΔO (μatoms) | ΔP (μmoles) | P : O |
|---|---|---|---|---|---|---|
| 0·52 | 15 | Glutamate | .. | 6·5 | 10·1 | 1·5 |
| | | | (No NAA) | 6·3 | 9·9 | 1·5 |
| 0·56 | 20 | Glutamate | .. | 6·6 | 11·9 | 1·8 |
| | | | Hex-G | 5·8 | 14·5 | 2·5 |
| 0·61 | 15 | Succinate | .. | 3·6 | 2·9 | 0·8 |
| 0·94 | 15 | Glutamate | .. | 6·6 | 7·9 | 1·2 |
| | | Succinate | .. | 4·4 | 6·5 | 1·4 |
| 0·47 | 15 | Glutamate | Hex-G | 4·7 | 10·8 | 2·2 |
| | 20 | Succinate | .. | 3·4 | 3·4 | 1·0 |
| 0·67 | 15 | Glutamate | (No DPN) | 1·1 | 2·2 | 2·0 |
| | | | (No DPN)* | 1·2 | 2·5 | 2·1 |
| | | | .. * | 4·4 | 7·9 | 1·8 |
| 0·60 | 30 | Pyruvate | (No DPN)* | 2·4 | .. | .. |
| | 60 | | (No DPN)* | 4·2 | .. | .. |
| | 30 | | .. * | 4·2 | .. | .. |
| | 60 | | .. * | 7·6 | .. | .. |
| | 60 | | EDTA (No DPN)* | 7·8 | .. | .. |
| 0·80 | 60 | Pyruvate | (No DPN)†, * | 7·8 | .. | .. |
| | | | .. †, * | 7·9 | .. | .. |
| 0·50 | 20 | Glutamate | (No DPN)* | 2·6 | 3·2 | 1·2 |
| | | | .. * | 9·4 | 14·1 | 1·5 |
| | | | EDTA (No DPN)* | 5·3 | 10·6 | 2·0 |
| 0·47 | 20 | Glutamate | (No DPN)†, * | 4·8 | .. | .. |
| | | | .. †, * | 6·7 | .. | .. |
| *Rat liver*— | | | | | | |
| 0·68 | 15 | Glutamate | (No DPN) | 6·5 | 16·3 | 2·5 |
| | | Glutamate | .. | 7·0 | 15·5 | 2·2 |
| | | Succinate | .. | 7·0 | 11·9 | 1·7 |

1957*a*) was found to raise the $O_2$ consumption of the hepatoma particles both with glutamate and pyruvate as the substrates, when it was included in the isolation medium in a concentration of 0·01 instead of 0·001 M or in the final incubation medium (0·005 M) in which mitochondria prepared in 0·001 M EDTA were present.* The latter finding indicated that in the absence of sufficient EDTA the release of DPN from the mitochondria occurred very early during the incubation of the particles at 27° C.

* Since 0·3 ml. of this suspension was added to the flasks to give a final volume of 1·6 ml., 0·00019 M EDTA was finally present. This concentration was apparently too low to counteract the release of DPN from the mitochondria (binding of $Ca^{2+}$ ?), although it had been active in this respect with mitochondria from spontaneous mouse hepatomas (Emmelot and Bos, 1957*a*).

*Succinate oxidation.*—As shown in Table VIII the oxidation of succinate was less than that of glutamate. The P : O ratio obtained with succinate in the absence of glucose hexokinase varied from 0·8–1·4, that is 40–70 per cent of the theoretical value. The average oxygen consumption in the presence of succinate amounted to 24 $\mu$atoms O/mg. N/hour for BY 252 rat hepatoma and to 45 $\mu$atoms O/mg. N/hour for rat liver mitochondria.

FIG. 14.—*Oxidation of hexanoate by BY 252 rat hepatoma mitochondria.*

Mitochondria isolated in 0·25 M sucrose containing $10^{-3}$ M EDTA; 0·66 mg. mitochondrial N present in the flasks.

Incubation as described (Emmelot, 1957*a*); hexanoate (2 $\mu$moles), L-malate (0·0003 M), ATP (0·0007 M) and DPN (0·001 M) present in addition.

Oxygen consumption corrected for that obtained in the absence of hexanoate.

*Fatty acid oxidation.*—It has further been found that the BY 252 hepatoma mitochondria, isolated in 0·25 M sucrose plus 0·001 M EDTA, may be capable of oxidizing hexanoate in the presence of a catalytic amount (0·0003 M) of L-malate. A marked lag period during the first 30 minutes of incubation was observed (Fig. 14). This phenomenon suggests that during the initial period of incubation the mitochondria lack a sufficient concentration of a critical factor that has to be built up before oxidation can proceed at a maximal rate. In some of the early experiments the mitochondria from transplants, which had been growing for 10 days or even longer, were apparently unable to overcome this lag phase, and oxygen consumption proceeded only very slowly during the whole period of incubation. The nature of the limiting factor is under further study; so far addition of ATP, coenzyme A, DPN, flavineadenine dinucleotide or nicotinamide has been without result. Octanoate dissolved in a 5 per cent serum albumin solution and added to the flasks (2 $\mu$moles fatty acid, final concentration albumin 0·3 per cent) was also oxidized after a lag period of low $O_2$-uptake. In the latter case only 11 $\mu$l $O_2$ were consumed in the first 30 minutes, whereas 34, 50, 58 $\mu$l $O_2$ were taken up in each following half hour interval, so that after 4 hours a total of 440 $\mu$l $O_2$ was consumed after which the respiration slowed down (1·02 mg mitochondrial N/flask; 9 days' transplant). In more recent experiments carried out with mitochondria from 6–7 day-old transplants, hexanoate (no albumine) was oxidized very vigorously since, for instance, 18, 25, 34, 43, 44 and 50 $\mu$l. of oxygen were consumed during successive 10 minute intervals (total 214 $\mu$l. $O_2$ in 60 minutes, 0·82 mg. N). In the presence of octanoate, added as albumin complex, 210 $\mu$l. $O_2$ were consumed in 120 minutes (0·62 mg. N; 47,60 and 103 $\mu$l. $O_2$ in the three successive 40 minute intervals) and in the presence of $\beta$-hydroxybutyrate (24 $\mu$moles, no albumin) 240 $\mu$l. $O_2$ (0·73 mg. N; 45, 75 and 125 $\mu$l. in three successive 40 minute intervals) were taken up.

When oxidation proceeded at its best, 33, 22 and 23 $\mu$atoms oxygen were consumed/mg. N/hour, in the presence of hexanoate, octanoate and $\beta$-hydroxybutyrate, respectively. The function to oxidize the three fatty acids was apparently dependent upon the physiological state of the tumour transplants since the best results were obtained with small transplants which had been grown for maximally 7 days after grafting. A lag phase in the $O_2$ consumption of the tumour mitochondria, in contrast to normal rat and mouse liver mitochondria, was, however, always observed.

The latter oxidized palmitate (0·4 $\mu$mole), added singly or as an albumin complex, but we have as yet observed no definite oxidation of this substrate by the BY 252 hepatoma mitochondria.

*Respiration of slices.*—The endogenous respiration of *slices* of intraperitoneal transplants of the BY 252 rat hepatoma was similar to that of livers of the strain R-Amsterdam rat. Glucose always inhibited the respiration of the hepatoma tissue (Crabtree effect; Crabtree, 1929) but, like pyruvate, stimulated that of liver (Table IX). In a number of experiments glucose had no effect on liver respiration. Pyruvate did not affect the oxygen consumption of the hepatoma. Succinate stimulated liver respiration approximately 3-fold but had a much smaller effect in the case of the hepatoma (1·5-fold stimulation).

TABLE IX.—*Respiration by slices of the BY 252 rat hepatoma and of rat liver*

Fifteen mg., respectively 18 mg., dry weight slices incubated in 1·6 ml. Krebs-Ringer phosphate (0·02 M) buffer of pH 7·4 at 37° C. Twenty $\mu$moles glucose, pyruvate and succinate added as indicated.

| | Microatoms oxygen consumed after minutes | | | |
|---|---|---|---|---|
| | *BY* 252 *hepatoma* | | *Rat liver* | |
| Addition | 60 | 90 | 60 | 90 |
| None | 4·9 | 7·2 | 5·8 | 8·3 |
| Glucose | 3·9 | 6·2 | 7·0 | 10·7 |
| Pyruvate | 5·0 | 7·6 | 6·7 | 10·0 |
| Succinate | 7·2 | 11·2 | 16·2 | 24·0 |

*Primary rat and primary and transplanted mouse hepatomas: DPN-linked oxidations.*—Mitochondria prepared from primary DAB-induced rat hepatomas of the solid type also oxidized glutamate in the presence of DPN. Succinate was oxidized by these mitochondria at a similar or faster rate than glutamate. Mitochondria from subcutaneous transplants of four different mouse heptomas which originated five or more years ago in the livers of female CBA mice either spontaneously (tumours T 26473 and T 28012) or following the administration of AAT (tumours CBA 71 and CBA 80) were likewise active. For pyruvate oxidation compare below and Emmelot and Bos (1957*a*).

*Fatty acid oxidation of liver and hepatoma mitochondria from rats fed DAB.*—Mitochondria from livers of rats fed DAB (hepatomas removed or not yet present) oxidized octanoate regardless of the fact whether the livers showed severe or mild steatosis and/or were affected by cirrhosis or other pathological changes (Table X); it was not necessary to add DPN or a primer of fatty acid oxidation. These results indicate, first, that the fatty infiltration was not due to a diminished capacity of the neoplastic liver mitochondria to oxidize the endogenous fatty acids and, secondly, that these particles contained enough endogenous substrate and DPN to generate ATP for the initial conversion of octanoate to octanoyl-coenzyme A, and for the fatty acid oxidation *per se*. Mitochondria from the " hyperplastomas " oxidized octanoate and $\beta$-hydroxybutyrate but these particles were more or less dependent upon added DPN for full activity. The primary rat hepatoma mitochondria as a rule did not oxidize octanoate (Emmelot and Bos, 1957*a*); the oxidation that has been noted in a number of cases was, however, comparable in rate to that of normal liver mitochondria so that these positive results were not likely to be due to the presence of normal liver cells in the particular hepatoma. An uptake of oxygen in the presence of DL-$\beta$-hydroxybutyrate could be obtained regularly. In those cases in which oxidation of octanoate could not be achieved and in which the oxidation of $\beta$-hydroxybutyrate was small (approximately 3 $\mu$atoms of oxygen taken up in 60 minutes under special conditions, compare Emmelot and Bos, 1957*a*) the histological diagnosis was invariably malignant solid (adeno or mixed) hepatoma. Positive octanoate and moderate to high $\beta$-hydroxybutyrate oxidation was obtained with mitochondria which appeared to be isolated from solid trabecular hepatomas which resembled liver tissue, though some mitochondrial preparations of such tumours were not so active. In mitochondrial preparations of two of the tumours of the latter type extremely low free ATPase activity has been found.

*Fatty acid oxidation of liver and hepatoma mitochondria from mice fed AAT.*—The various stages of the neoplastic process have also been followed in livers of mice receiving AAT (Table XI). Before macroscopic lesions were present octanoate oxidation was diminished because endogenous citric acid cycle intermediates, which through the oxidative generation of ATP must prime fatty acid oxidation, were apparently missing. This was concluded from the fact that $\alpha$-oxycaproate or L-malate had to be added as a primer in these experiments in order to obtain oxidation. The mitochondria from these livers were more labile than normal mouse liver mitochondria since washing with a KCl-phosphate buffer, instead of isotonic sucrose, destroyed the ability of the former, but not of the latter, particles to oxidize octanoate. As soon as pathological changes were visible the liver particles needed added DPN for activity (Emmelot, 1954). The requirement for DPN and primer in the octanoate oxidation was not observed

TABLE X.—*Oxidative properties of mitochondria from pre-neoplastic liver, hyperplastomas and hepatomas of rats fed DAB*

α-OC stands for α-oxycaproate, *S* for 0·25 M sucrose, *s* + *v* for 0·25 M sucrose containing 0·001 M EDTA and BHB for β-hydroxybutyrate. The substrates marked † have been incubated in the presence of 0·0003 M L-malate to ensure complete oxidation of the $C_2$ fragments ; α-OC or L-malate were present to prime octanoate oxidation. Conditions : Emmelot and Bos (1955, 1957*a*). The oxygen-uptake in the presence of octanoate plus α-OC or L-malate (and DPN) has been corrected for that obtained in the presence of α-OC or L-malate (and DPN) alone. Time of incubation 45–60 minutes.

| Mitochondria from | Isolation medium (mg. mitochondrial N/flask) | Substrate | Oxygen uptake ($mm.^3$) DPN absent | DPN present |
|---|---|---|---|---|
| Liver, DAB 10 months, hepatoma removed (steatosis, local hyperplasia) | S (0·81) | Octanoate | 57 | .. |
| | | Octanoate + α-OC | 84 | 131 |
| Liver, DAB 10 months, no hepatoma (steatosis, local hyperplasia, cirrhosis) | S (0·97) | Octanoate | 87 | .. |
| | | Octanoate + α-OC | 84 | 112 |
| Hyperplastoma | S (1·08) | Octanoate | 20 | .. |
| | | Octanoate + α-OC | 18 | 111 |
| Corresponding liver (severe steatosis, no necrosis or cirrhosis) | S (1·53) | Octanoate | 120 | .. |
| | | Octanoate + α-OC | 112 | 150 |
| Hyperplastoma | S (1·78) | Octanoate | 71 | .. |
| | | Octanoate + α-OC | 65 | 177 |
| Corresponding liver (mild steatosis, some regeneration, glycogen normal) | S (1·38) | Octanoate | 93 | .. |
| | | Octanoate + α-OC | 107 | 129 |
| Hyperplastoma | s + v (1·28) | Octanoate† | .. | 121 |
| Hyperplastoma | s + v (0·82) | DL-BHB | .. | 108* |
| Hyperplastoma mixed with solid hepatoma | s + v (1·18) | DL-BHB† | .. | 130 |
| | | Pyruvate† | .. | 127 |
| Solid trabecular hepatoma, resembling liver | s + v (1·21) | DL-BHB | .. | 84 |
| | | Octanoate† | .. | 105 |
| Solid (malignant) hepatoma | s + v (1·42) | DL-BHB† | 6 | 33** |
| | | Octanoate† | 0 | 0** |
| | | Pyruvate† | 0 | 95** |
| Solid (malignant) hepatoma | s + v (1·08) | Pyruvate† | .. | 113 |

* 5·6 μmoles acetoacetate produced.
** Extra ATP added from side arm of respirometers (compare Emmelot and Bos, 1957*a*).

with the corresponding rat liver mitochondria which may indicate that the latter particles were more intact. The primary mouse hepatoma particles (Table XI) did not oxidize L(+)-β-hydroxybutyrate and octanoate but remained capable of oxidizing the D(−)isomer of β-hydroxybutyrate and pyruvate.

*Fatty acid oxidation of spontaneous and transplanted mouse hepatoma mitochondria.*—The different abilities of mitochondria isolated from these tumours to oxidize fatty acids has been described earlier (Emmelot and Bos, 1955, 1957*a*).

*Oxidative phosphorylations.*—The free ATPase activities of the mitochondria from the various tumours depressed the efficiency of the oxidative phosphory-

TABLE XI.—*Oxidative properties of mitochondria from pre-neoplastic, neoplastic livers and hepatomas of CBA mice fed AAT*

Abbreviations as used in Table X ; (KCl-P) indicates that the second washing of the mitochondria has been carried out with KCl-phosphate buffer instead of sucrose. Liver plus adhering hepatoma has been used in the first 10 experiments.

| Mitochondria from | Isolation | Amount of mitochondria (mg. N/flask) | Substrate | Oxygen uptake (mm.$^3$) DPN absent | Oxygen uptake (mm.$^3$) DPN present |
|---|---|---|---|---|---|
| Livers of controls (1 year old) | S | 1·34 | Octanoate | 120 | .. |
| | | | Octanoate + α-OC | 112 | .. |
| | S(KCl-P) | 1·34 | Octanoate | 82 | .. |
| | | | Octanoate + α-OC | 130 | .. |
| 3 livers, AAT 8 mo ("normal") | S | 1·21 | Octanoate | 10 | .. |
| | | | Octanoate + α-OC | 93 | .. |
| 2 livers, AAT 9 mo ("hypertrophic") | S | 1·80 | DL-BHB | 97 (6·6)* | 92 |
| 3 livers, AAF 12 mo (neoplastic foci) | S | 1·38 | Octanoate† | .. | 258 |
| | S(KCl-P) | 1·38 | Octanoate† | .. | 16 |
| 3 livers, AAT 10 mo (neoplastic foci) | S | 1·36 | Octanoate† | 5 | 109 |
| 3 livers, AAT 11 mo (one small hepatoma) | S | 1·25 | Choline | 74 | 108 |
| 3 livers, AAT 11 mo (many. small hepatomas) | S | 1·11 | Octanoate | 4 | 13 |
| | | | Octanoate + α-OC | 3 | 106 |
| 11 livers, AAT 12 mo small and large hepatomas (2 separate experiments) | S | 1·30 ; 1·80 | D-BHB | 0 ; 0 | 91 ; 76 |
| | | | L-BHB + α-OC | 0 ; 0 | 0 ; 0 |
| 3 livers, AAT 13 mo (large hepatomas) | S | 1·51 | DL-BHB | 0 | 77 (6·9)* |
| 7 livers, AAT 13 mo (small and large hepatomas) | S | 1·80 | Octanoate† | .. | 110 (0·5)* |
| 3 hepatomas, with small amount of adhering liver | S | 1·19 | Octanoate + α-OC | 16 | 137 |
| 3 hepatomas, dissected from 2 livers | S | 1·09 | Octanoate | 3 | 8 |
| | | | Octanoate + α-OC | 5 | 15 |
| Hepatomas from 11 livers (two separate experiments) | s + v | 1·71 ; 1·23 | DL-BHB | 0 ; 5 | 83 ; 106 (7·2)* |
| | | | Octanoate† | 5 ; 3 | 7 ; 4 |
| | | | Pyruvate† | .. ; .. | 105 ; 124 |
| Hepatomas from 3 livers | s + v + NAA | 1·03 | L-Glutamate‡ | 13 | 84 |

* μMoles acetate produced.
† In the presence of 0·0003 M L-malate, corrected for the oxygen in the presence of malate alone.
‡ Incubated during 20 minutes.

lations when incubation was carried out in the absence of the ATPase inhibitor fluoride and the ATP-trapping system glucose-hexokinase (Table XII). It was therefore not surprising that the rate of oxidation of these particles was much less dependent upon added phosphate and ADP than in the case of normal liver mitochondria. Addition of fluoride or glucose-hexokinase markedly favoured the P : O ratios. Similar effects have been demonstrated with liver mitochondria damaged by mild ageing or treatment with electrolyte. A more satisfactory

TABLE XII.—*Oxidative phosphorylation by tumour mitochondria ; effect of hexokinase on the P : O ratio*

Mitochondria isolated in 0·25 M sucrose containing $10^{-3}$ M EDTA ($s + v$) or in raffinose-dextran-heparine-EDTA ($r + d$). Incubation as described (Emmelot, 1957*a*).

Glu = glutamate, Succin. = succinate, G-Hex. = glucose and hexokinase.

| Mitochondria from tumour | Isolation medium | Substrate | Addition | ΔP μmoles, N/20 min. | ΔO resp. μatoms/mg. | P : O |
|---|---|---|---|---|---|---|
| T 26473 | s + v | Glu | .. | 6·3 | 7·0 | 0·9 |
| | | | G-Hex. | 12·6 | 7·0 | 1·8 |
| T 28012 | s + v | Glu | .. | 7·3 | 7·3 | 1·0 |
| | | | G-Hex. | 14·4 | 7·2 | 2·0 |
| | s + v | Succin. | .. | 6·6 | 7·3 | 0·9 |
| | | | KF($10^{-2}$M) | 9·4 | 6·1 | 1·5 |
| Primary rat hepatomas (solid type) | s + v | Glu | .. | 6·3 | 6·3 | 1·0 |
| | | | G-Hex. | 11·6 | 6·4 | 1·8 |
| | r + d | Glu | .. | 9·0 | 6·0 | 1·5 |
| | | Succin. | .. | 10·8 | 9·0 | 1·2 |
| $S_3A$ ascites carcinoma | s + v | Glu | .. | 7·5 | 5·0 | 1·5 |
| | | | G-Hex. | 11·8 | 5·1 | 2·3 |
| UV256 sarcoma | s + v | Glu | .. | 4·2 | 4·2 | 1·0 |
| | r + d | Glu | .. | 6·2 | 4·1 | 1·5 |
| | r + d | Glu | .. | 10·4 | 4·7 | 2·2 |
| | | Succin. | .. | 12·6 | 8·4 | 1·5 |

P : O ratio with primary rat hepatoma and sarcoma mitochondria could also be obtained by isolating the particles in the raffinose dextran-heparine-EDTA medium, which is said to protect the structure of mitochondria (Birbeck and Reid, 1956).

*Is " lability " an exclusive property of isolated tumour mitochondria ?*—All the present results indicate that the integrated enzymic functions of the tumour mitochondria were less intact than those of normal liver mitochondria. Apart from the possibility to induce a similar change in isolated liver mitochondria by noxious treatment such as ageing or freeze-thawing, isolated mitochondria from pathological livers other than neoplastic, may also show an enhanced lability. Dianzani (1954, 1955), for instance, observed a loss of DPN and reduced P : O ratios with mitochondria from experimental fatty livers. We have encountered high free ATPase activity in liver mitochondria from normal Odz × dz hybrid mice (Emmelot *et al.*, 1956) ; these particles were in many cases unable to oxidize octanoate. DPN addition has been found necessary to obtain fatty acid oxidation by mitochondria isolated from the liver of two-year old $O_{20}$ and CBA mice (Emmelot and Bos, 1955) and glutamate oxidation by liver mitochondria from apparently healthy rats (Kielley, 1957). It should be noted, however, that the enhanced lability was not inherent to the liver mitochondria of the mice (strain CBA) and rats (strain R-Amsterdam) with which the present and earlier experiments were carried out.

*Guinea-pig liver mitochondria.*—A marked lability has also been observed in mitochondria from livers of guinea-pigs of a partly inbred colony kept in our Institute. Sucrose mitochondria prepared from these livers showed no detectable glucose-6-phosphatase activity and since the microsomal fraction contained a high level of the latter enzyme, it was concluded that the mitochondria were practically devoid of microsomal contamination. Marked free ATPase activities were frequently encountered in the mitochondrial preparations, and DNP stimulated the ATP-dephosphorylation of the guinea pig liver mitochondria to a smaller extent than the corresponding process of mouse and rat liver mitochondria. Glutamate was oxidized at a high rate only when DPN was added ; in the absence of the coenzyme only 10–20 per cent of the oxygen uptake of the DPN-fortified particles was noted. Isolation of the particles in the presence of 0·001 M EDTA did not improve this situation, but after isolation in 0·25 M sucrose containing 0·01 M EDTA, the (higher) oxygen consumption (Table XIV) was only slightly stimulated by DPN.

*The effect of thyroxine on the oxidative phosphorylation of hepatoma and guinea-pig liver mitochondria.*—Thyroxine has been found to be without effect on the oxidative phosphorylation of freshly-prepared liver mitochondria from the rat (Martius and Hess, 1955 ; Klemperer, 1955). Only after a pretreatment of the particles, such as a preliminary incubation with thyroxine at 0° C or suspension in a hypotonic medium (Tapley, Cooper and Lehninger, 1955) does the hormone uncouple the oxidations from the accompanying phosphorylations in the respiratory chain. This has been taken to mean that some time is needed for thyroxine to penetrate into the mitochondria via the intact membranes of fresh liver particles. The oxidative phosphorylations of fresh hamster liver mitochondria, washed once with electrolyte instead of sucrose, have been found by Hoch and Lipmann (1954) to be readily uncoupled by thyroxine without further pretreatment and these particles were, therefore, considered to be " leaky ". However, the procedures of pretreatment used to bring about the uncoupling effect are not only noxious to the mitochondrial structure but also to its integrated enzymic performance, which is closely related to structure. The observation that the thyroxine-sensitive hamster liver mitochondria readily loose DPN and cytochrome *c* whereas the insensitive rat liver mitochondria do not, fits in not only with the " leakiness " but also with a reduced overall functional intactness of the particles. Whatever the explanation may be, it is evident that damaged mitochondria (compare Table XIII, 1st experiment) do respond readily in terms of uncoupling of their oxidative phosphorylation to thyroxine. It would therefore be of interest to study the effect of thyroxine on the oxidative phosphorylations of these hepatoma mitochondria which give evidence of an increased lability as compared with fresh liver mitochondria. Previous experiments have shown that thyroxine may indeed uncouple the oxidative phosphorylations of fresh untreated tumour mitochondria (Emmelot and Brombacher, 1957). In the present experiments the phosphorylations accompanying the oxidation of glutamate by fresh liver mitochondria from our rats were sometimes slightly depressed by thyroxine ($7{\cdot}5 \times 10^{-5}$ M), *i.e.* 25 per cent at its most, whereas a drop of 40–60 per cent in the P : O ratio of the BY 252 rat hepatoma mitochondria was consistently noted. The oxidative phosphorylations of fresh mouse liver mitochondria were not affected by thyroxine. Neither did thyroxine affect the P : O ratios obtained with mitochondria from the transplanted mouse hepatoma CBA 71. The phosphorylations accompanying the oxidation of $\beta$-hydroxybutyrate of T 28012 mitochondria were not affected, but with glutamate as substrate uncoupling in the presence of thyroxine was noted. The hormone markedly depressed the oxidative phosphorylation of T 26473

TABLE XIII.—*Effect of thyroxine on the oxidative phosphorylation of hepatoma mitochondria*

Mitochondria isolated in 0·25 M sucrose containing $10^{-3}$ M EDTA. 0·5–0·9 mg. mitochondrial N present in the various experiments.
In all experiments freshly isolated mitochondria were used, except in one case in which liver mitochondria were sonicated during 5 minutes prior to incubation. Glutamate (GLU) oxidation in histidine buffer, as described previously (Emmelot, 1957*a*). β-Hydroxybutyrate (BHB) and pyruvate oxidation in phosphate buffer (Emmelot and Brombacher, 1957). DPN added in a final concentration of $10^{-3}$ M. Thyroxine ($7·5 \times 10^{-5}$ M) present in the main compartment except in two experiments marked *, in which the hormone was added from the side arm of the respirometer flasks after the time allowed for temperature equilibration. KF ($10^{-2}$ M), EDTA ($10^{-3}$ M) and glucose-hexokinase were added as indicated. Incubation during 15–20 minutes at 27° C ; pH 7·4.

| Mitochondria from | Substrate | Addition | Thyroxine | ΔP (μmoles) | ΔO (μatoms) | P : O |
|---|---|---|---|---|---|---|
| Rat liver | GLU | .. | – | 14·2 | 6·7 | 2·1 |
| | | | + | 11·1 | 6·5 | 1·7 |
| | | (Sonicated mitochondria) | – | 13·4 | 7·8 | 1·7 |
| | | | + | 1·5 | 6·4 | 0·2 |
| BY 252—rat hepatoma | GLU | .. | – | 6·9 | 4·3 | 1·6 |
| | | | + | 3·0 | 3·2 | 0·9 |
| | GLU | .. | – | 8·7 | 5·8 | 1·5 |
| | | | + | 1·7 | 2·8 | 0·6 |
| | | $10^{-3}$ M EDTA | – | 10·5 | 7·0 | 1·5 |
| | | | + | 5·1 | 4·1 | 1·2 |
| CBA71 — mouse hepatoma | Pyruvate (L-malate) | Fluoride, G-Hex. | – | 10·8 | 5·4 | 2·0 |
| | | | + | 9·8 | 5·4 | 1·8 |
| | GLU | Fluoride | – | 4·8 | 3·6 | 1·3 |
| | | | + | 3·6 | 2·6 | 1·4 |
| T 26473 — mouse hepatoma | BHB | Fluoride, G-Hex. | – | 5·4 | 2·0 | 2·7 |
| | | | +* | 3·0 | 2·5 | 1·2 |
| | BHB | Fluoride, G-Hex. | – | 5·1 | 2·4 | 2·1 |
| | | | +* | 1·5 | 2·0 | 0·75 |
| | BHB | Fluoride, G-Hex. | – | 6·0 | 2·5 | 2·4 |
| | | | + | 1·9 | 2·1 | 0·9 |
| | GLU | Fluoride | – | 5·8 | 4·3 | 1·3 |
| | | | + | 0·7 | 2·2 | 0·3 |
| T 28012 — mouse hepatoma | BHB | Fluoride, G-Hex. | – | 7·7 | 3·7 | 2·1 |
| | | | + | 7·2 | 3·6 | 2·0 |
| | GLU | G-Hex. | – | 6·8 | 4·5 | 1·5 |
| | | | + | 2·7 | 4·9 | 0·5 |
| | GLU | Fluoride | – | 3·2 | 3·2 | 1·0 |
| | | | + | 0 | 1·6 | 0·0 |

mitochondria. Thyroxine was present in the main compartment of the respirometers in all except two experiments with T 26473 mitochondria in which the hormone was added from the sidearm together with the oxidizable substrate after temperature equilibration had been reached. This procedure was followed in order to reduce the contact between the particles and the hormone to a minimum. Since uncoupling still occurred under the latter conditions, it may be concluded that thyroxine acted momentarily. The reasons for the difference in effect of thyroxine on the various hepatoma mitochondria is not clear, there is some evidence that it may be connected with differences in the intrinsic lability of the particles.

It has been shown in an earlier paper (Emmelot and Bos, 1958) that thyroxine inhibited the oxidation of glutamate by mouse liver mitochondria but that in the presence of 0·001 M DPN hardly, if any, inhibition resulted. By contrast, thyroxine inhibited the glutamate oxidation of the tumour particles markedly in most of the present experiments in spite of the excess DPN being added. The latter phenomenon might be related to the weak binding between DPN and the hepatoma particles.

A marked inhibition of glutamate oxidation in the presence of thyroxine and DPN has also been noted with the guinea pig liver mitochondria, which, like the hepatoma and unlike the mouse and rat liver mitochondria, need DPN for optimal oxidation in the absence of thyroxine. The resemblance between fresh guinea pig liver and hepatoma mitochondria was the more striking since the oxidative phosphorylations of the former particles were also completely abolished by thyroxine (Table XIV). EDTA counteracted (compare Park *et al.*, 1956) the uncoupling of the oxidative phosphorylations of the guinea pig liver mitochondria (Table XIV), the BY 252 rat hepatoma mitochondria (Table XIII) and hypotonically-treated thyroxine-sensitive mouse liver mitochondria (Table XIV).

In summarizing the results of the experiments with thyroxine it may be stated that they lend additional support to the view that mouse and rat hepatoma mitochondria may be functionally less intact than the corresponding liver mitochondria, but that this property is not specific for the tumour mitochondria.

### DISCUSSION

Since in the present investigation mainly " solid " tumours (solid in the sense of a coherent mass of cells in contrast to the ascites form in which a tumour may be grown) have been used, it seems well to consider briefly the statement of Warburg (1956) that only ascites tumours should be studied and that " solid " tumours—and especially " solid " spontaneous tumours—need no longer be subjected to metabolic examinations to-day since the " solid " tumours are usually so impure histologically in that normal cells are present. Considered in regard to transplanted " solid " tumours, this statement seems to us to be incorrect for the following reasons. Host fibrous tissue (stroma) is present in all organoid formations whether tumour or not. However, in rapidly growing transplanted carcinomas the fibrous elements are less developed than in the slower growing primary tumour but not necessarily more than in the parent normal tissue. Continuous transplantation can be considered as constituting a means of obtaining the medullary—as opposed to the scirrhus—type of the " solid " carcinoma. Thus, as long as normal tissues, not grown in tissue culture, are used for metabolic studies, the use of transplanted

TABLE XIV.—*Effect of thyroxine on the oxidative phosphorylation of fresh guinea-pig liver and hypotonically-treated mouse liver mitochondria in the absence and presence of EDTA*

Glutamate oxidation in histidine buffer in the presence of DPN ($10^{-3}$ M) as described previously (Emmelot, 1957*a*). 0·005 M and 0·0025 M $MgCl_2$ were added, respectively, in the experiments with the guinea-pig liver and the hypotonically-treated mouse liver mitochondria. The latter were obtained by suspending fresh mouse liver mitochondria in 0·075 M sucrose during 10 minutes at 0° C prior to use. EDTA added to the respirometers or included in the isolation medium as indicated. 0·3 Ml. of the mitochondrial suspensions corresponding to 250–300 mg. of wet weight liver were added to give a final volume of 1·6 ml. S stands for 0·25 M sucrose.

| Mitochondria from | Isolation | Addition | Thyroxine | ΔP (μmoles) | ΔO (μatoms) | P : O |
|---|---|---|---|---|---|---|
| Guinea-pig liver | S | .. | − | 10·2 | 6·7 | 1·5 |
| | | | + | 2·0 | 4·3 | 0·5 |
| | | G-Hex. | − | 12·3 | 5·8 | 2·1 |
| | | | + | 2·4 | 4·5 | 0·5 |
| Guinea-pig liver | S | .. | − | 9·8 | 6·1 | 1·6 |
| | | | + | 0·0 | 3·8 | 0·0 |
| | S + $10^{-3}$ M EDTA | .. | − | 12·6 | 5·9 | 2·1 |
| | | | + | 1·5 | 3·7 | 0·4 |
| | S + $10^{-2}$ M EDTA | .. | − | 9·9 | 5·5 | 1·8 |
| | | | + | 8·0 | 5·0 | 1·6 |
| | S + $10^{-2}$ M EDTA | (No DPN) | − | 10·2 | 5·1 | 2·0 |
| | | | + | 5·0 | 2·5 | 2·0 |
| Guinea-pig liver | S | .. | − | 12·8 | 8·0 | 1·6 |
| | | | + | 0·0 | 4·9 | 0·0 |
| | | $10^{-3}$ M EDTA | − | 14·6 | 7·3 | 2·0 |
| | | | + | 8·8 | 5·5 | 1·6 |
| | | $10^{-2}$ M EDTA | − | 13·8 | 6·0 | 2·3 |
| | | | + | 9·8 | 4·2 | 2·3 |
| Mouse liver hypotonically treated | S | .. | − | 11·7 | 9·8 | 1·2 |
| | | | + | 1·4 | 7·0 | 0·2 |
| | | $10^{-3}$ M EDTA | − | 13·3 | 7·7 | 1·7 |
| | | | + | 10·0 | 6·2 | 1·6 |

medullary tumours is fully warranted if the latter are free from normal cells besides the stroma. Metabolic investigations on normal cells cultured *in vitro* should be considered in the light of the accumulating evidence that enzyme functions, present in the intact tissue, may be lost (Perske, Parks, and Walker, 1957). Now, although early transplant generations of solid tumours may occasionally contain some rests of normal tissue, most well-established transplanted tumours are free from the latter, as judged by all available histological criteria. We can safely state that this is the case with the BY 252 rat hepatoma and other transplanted tumours used in our investigations.

The fact remains, however, that the cell population of a transplanted tumour, in contrast to that of the ascites tumour, may not be uniform in that differences in chromosome number, metabolic activities and morphology between individual cells may exist to some extent (references in Klein, 1956, and Sylvén and Malmgren, 1957). These differences between the tumour cells may be due to their deoxyribonucleic acid-content (compare Kit and Griffin, 1958) but they may, partly at least, also be caused by mechanical factors which govern the supply of nutrients and oxygen and, subsequently, the synthesis of enzymic-active proteins. However, all these cells are *tumour* cells and the study of their (average) metabolic activities is justified since there is no evidence whatsoever to suggest that a more uniform cell population selected from the "solid" tumour by environmental pressure, as seems to be the case in ascites cell formation, represents the type of tumour cell as it originated in first instance. On the contrary, since most tumours are not convertible to the ascites form, one may assume that the particular requirements for this conversion are not met with in large cell populations which are definitely tumourous. Only the most anaplastic tumours are easily convertible, whereas others need several passages before complete transformation into the ascites form results (Klein, 1956). Physiological adaptation and mutational adjustments seem to be involved in the transformation. Moreover, ascites tumour cells, as well as *in vitro* cultured free cells, have lost their original relation to the host stroma, a situation which makes these cells *ipso facto* different from the original cells of the coherent tumour. The absence of such an organized architecture may result in a profound alteration of certain metabolic properties as, for instance, the active transport of metabolites into the cells. The study of ascites cells thus furnishes information about a very particular, but not about *the* cancer cell. In fact, to seek information about the metabolism of *the* tumour cell may be an illusory ideal, as demonstrated, for instance, by the varied responses of different tumours in the face of a chemotherapeutical challenge and by the increasing evidence of differences in the metabolic activities between various tumours (compare for ascites tumours: Forssberg and Révész, 1957; Purdom, Ambrose and Klein, 1958). Such differences among tumours may give information about the grade of malignancy, as exemplified biologically by the degree of differentiation, growth rate, potency to metastasize and biochemically by the loss of certain enzymic functions and the acquirement to capture and use preformed metabolites of the host to the advantage of metabolic channelling of precursors in alternative directions, cellular adhesiveness in relation to membrane charge (Purdom *et al.*, 1958), etc.

The ascites tumour furnishes a powerful tool for investigation of many highly interesting aspects of the cancer problem, but by its very nature the ascites tumour should not monopolize biochemical cancer research.

From the observations collected in this paper, which are mainly concerned with those biochemical properties dependent upon the integrated structural and enzymic functions of mitochondria, a number of conclusions can be drawn.

(i) Since our data concerning the mitochondrial glutamic dehydrogenase of the BY 252 rat hepatoma are in marked contrast to those published on the Novikoff hepatoma (Allard *et al.*, 1957), one arrives at the situation that two transplanted undifferentiated tumours of the same type and species differ qualitatively in an important enzyme. This represents an exception to the reverse of the principle of metabolic uniformity among undifferentiated tumours.

(ii) Sufficient evidence on differences in enzymatic activities between isolated mitochondria of various hepatomas is now available to conclude that no general statements about possible divergencies in mitochondrial activities can be derived from a comparative study of normal liver and one particular hepatoma unless such a conclusion is checked on several tumours of the same type. The same consideration may also apply to the metabolic activities of cellular structures other than mitochondria. It has been shown, for instance, that the microsomes in a 15,000 $\times$ g supernatant of the mouse hepatoma T 28102 are moderately active in incorporating radioactive leucine whereas those of T 26473 and of primary rat hepatomas were only very slightly active in this respect (Emmelot, 1957*b*). Very recently we have found a high incorporation of amino acid, resembling that obtained with liver preparations, in microsomes of a 15,000 $\times$ g supernatant of young, but not of old, transplants of the BY 252 rat hepatoma. It is interesting that the capability of the mitochondria from these tumours to oxidize fatty acids exactly parallels the amino acid incorporation studies, which suggests that the " lability " is common to both mitochondria and microsomes.

(iii) It has been shown that mouse and rat hepatoma mitochondria, as a class, tend to show an increased lability in comparison with the corresponding liver mitochondria. The lability of the particles manifests itself by a more or less ready loss of DPN from the particles (compare also Wenner and Weinhouse, 1953; Kielley, 1952), increased free ATPase activity in the presence of $Mg^{2+}$, decreased P : O ratios in the absence of fluoride and glucose-hexokinase, more exacting conditions for obtaining fatty acid oxidation (compare also Emmelot and Bos, 1955) and an abolishment of the oxidative phosphorylations of most of the fresh tumour particles in the presence of thyroxine. It should be emphasized that in several of these respects quantitative differences in the degree of variance from " normal " may be encountered among the mitochondria from various hepatomas (Emmelot and Bos, 1955; 1957*a*, *b*, *c*) and even among mitochondrial preparations from different transplants of the same hepatoma. As regards the latter aspect, it is suggested that necrobiotic phenomena might be involved in the case of the BY252 hepatoma.

Various observations suggest that the enhanced lability of the hepatoma mitochondria manifests itself *in vitro*. This applies, for example, to the loss of DPN from the BY 252 hepatoma particles. Moreover, direct analysis, carried out in the presence of NAA to prevent the enzymic destruction of DPN by DPNase, has shown that mitochondria from the primary hepatomas of our rats may contain from 25–50 per cent less DPN than normal rat liver mitochondria (compare also Glock and McLean, 1957) but that sometimes even no trace of DPN was present (assay with alcohol dehydrogenase). Since ascites tumour cells and tumour slices, including those of hepatomas, do oxidize fatty acids, we assume that the loss of fatty acid oxidation observed with certain mitochondrial preparations is not due to the absence of the necessary enzymes but to the damage inflicted upon the integrated function of the particles *in vitro*. Moreover, it has been shown that tumour mitochondria isolated in 0·25 M sucrose, unlike liver particles, do not oxidize octanoate, but that after isolation in 0·25 M sucrose containing 0·001M EDTA vigorous oxidation ensues (Emmelot and Bos, 1955). The experiments on the fatty acid oxidation of the BY 252 rat hepatoma mitochondria are another point in case. It should be noted that the reported absence of glutamic dehydrogenase activity from the Novikoff hepatoma mitochondria is not likely to be due to the lability of these particles. Disruption of the particles would bring this enzyme, which is rather stable, in solution ; Allard *et al.* (1957) were, however, unable to demonstrate enzyme activity in homogenates of the Novikoff hepatoma. What has been said about the hepatoma mitochondria also applies to other tumour mitochondria (e.g. Emmelot and Brombacher, 1956) but it should be emphasized that lability is not an exclusive property of isolated mitochondria from neoplastic tissues.

(iv) Apart from a quantitative or qualitative difference in enzyme function as a result of the greater lability of the isolated hepatoma mitochondria, such particles may also possess a smaller content of certain enzymes than liver mitochondria. This applies, for example, to the $Mg^{2+}$-activated ATPase of the sonic disrupted and to the DNP-activated ATPase of intact liver and tumour mitochondria. The possibility may, as yet, not be excluded that an inhibitor of the ATPase is present in the tumour mitochondrial preparations. In the former case disorganization of the structure has been induced deliberately in order to uncover the potential content of the enzyme. In a homogenate of the Novikoff hepatoma the succinoxidase activity is reduced to 13 per cent of the liver level (Novikoff, 1957). Since Allard *et al.* (1957) found that the number of mitochondria present in the latter tumour amounted to 25 per cent of that present in liver, one may conclude that the average Novikoff mitochondrion contains one-half of the succinic oxidase activity of a liver mitochondrion. Our data on the BY 252 rat hepatoma and those of Schneider and Hogeboom (1950) on a transplanted mouse hepatoma are in accordance herewith. Since the succinic oxidase does not appear to be easily inactivated by *in vitro* conditions, the above observation may be interpreted as reflecting the actual mitochondrial content of the enzyme. Since succinate oxidation is less dependent upon ATP turnover than the oxidation of other citric acid-cycle intermediates, the oxygen consumption of tumour slices in the presence of succinate may give a rough measure of the succinoxidase activity of intact cells. The much greater stimulation of the oxidation of liver slices by succinate over the no substrate level as compared with that of primary (Olson, 1951) and of transplanted (Table IX) rat hepatomas indicates that a marked difference in succinoxidase content of the normal and neoplastic tissues does exist. This is due to differences in the amount of mitochondria present and in the succinoxidase content per mitochondrion. It should, however, be pointed out that the lower succinoxidase activity of the hepatomas does apparently not function as a bottle-neck in the endogenous (fatty acid oxidation ; Medes, Paden and Weinhouse, 1957) respiration nor in the oxidation of carbohydrates, probably because other reactions (e.g., phosphorylation) are rate-limiting under these conditions.

(v) It has been shown that isolated mitochondria from " hyperplastomas ", solid trabecular hepatomas resembling liver tissue and malignant hepatomas (solid and adeno) of the rat show a decreased functional intactness in the order given. It is known that a close correspondence between mitochondrial morphology and function exists in that structural disorganization leads to impaired function. Of the mitochondrial enzymes the glutamic dehydrogenase is located in the soluble part (matrix) of the particles whereas most Krebs-cycle and phospho-transferring enzymes are located in the insoluble part (membranes). Our primary rat hepatomas are being studied by Professor van Rijssel and Dr. de Man at Leiden University with the electron microscope (de Man *et al.*, 1959). It has been found that the solid hepatoma mitochondria had a very heterogeneous appearance both in dimensions and fine-structure. In general the hepatoma mitochondria were smaller and present in lesser amount than liver mitochondria. Sometimes no internal membranes were seen but in other cases more " cristae " were present than in liver mitochondria. Particles of an obviously normal type and giant types were also encountered. Liver mitochondria contain an evenly dense matrix but in the hepatoma mitochondria sometimes very little matrix was present which was unevenly distributed thus giving rise to optically-empty cavities of an irregular and faded shape. It has been shown that enlarged and apparently degenerative mitochondrial structures next to small, more or less intact ones, are also present in the Novikoff hepatoma and other tumours (Howatson and Ham, 1955, 1957; Novikoff, 1957; Bernhard, 1957, 1958).

Considering the close correlation between mitochondrial structure and enzymic function, it is not difficult to envisage, first, that the enzyme content of liver and hepatoma mitochondria may vary in certain respects and, secondly, that the loss or inactivation of certain mitochondrial enzymes and co-factors *in vitro* may be due to the labile nature of at least part of the mitochondrial population of the hepatoma cells, as is suggested by the degenerative features in the fine-structure of these particles. These considerations may possibly also apply to other tumours.

What effect the observed change in fine-structure may have upon the activity of the tumour mitochondria *in situ* is not known. In so far as the mitochondrial oxidative phosphorylations are concerned no serious defects seem to result since the available evidence strongly suggests that the latter processes do occur in intact tumour cells. Addition of the uncoupling agent DNP to tumour slices or ascites cells leads to a marked rise in the oxygen consumption, an inhibition of certain metabolic reactions and to the abolishment of the Pasteur effect (Emmelot and van Vals, 1957*b*; Emmelot and Bos, unpublished).

### SUMMARY

1. A transplanted rat hepatoma of the solid (hepatocellular) type—the BY 252 hepatoma—has been established. Mitochondria were isolated from this tumour and their properties compared with those of normal liver mitochondria.

The ATPase activity of the fresh hepatoma particles was not latent; DNP activated the ATPase in the presence of $Mg^{2+}$ at 27° C but at 37° C hardly, if any, effect was noted; the ATPase of the tumour particles was already more or less completely active at the latter temperature in the presence of $Mg^{2+}$ alone. By contrast, most of the ATPase complement of normal liver mitochondria from rats of the same strain as that in which the hepatoma had arisen, was still in a latent state at 37° C. The total ATPase content, as manifested in fresh mitochondria by $Mg^{2+}$ plus DNP or in disrupted mitochondria by $Mg^{2+}$ alone, was smaller in the case of the hepatoma than of the liver. DNP abolished the Pasteur effect in slices of BY 252 hepatoma and other tumours (primary rat hepatomas, a mammary ascites carcinoma and a sarcoma of the mouse). DNP also activated the ATPases of the mitochondria isolated from the latter tumours.

2. The endogenous respiration of slices of the BY 252 hepatoma was not different from that of normal rat liver. Glucose inhibited the respiration of the hepatoma (Crabtree effect). Succinate stimulated the respiration of the hepatoma for about 50 per cent but that of liver for about 300 per cent.

Oxidation and phosphorylation by the BY 252 hepatoma mitochondria were also studied. The succinoxidase activity was about half of that shown by the same amount of normal rat liver particles. The hepatoma particles possessed a considerable glutamic dehydrogenase activity, in contrast to what is reported about the Novikoff rat hepatoma. The BY 252 hepatoma mitochondria released part of their DPN-complement during incubation. Oxidative phosphorylation in the absence of glucose-hexokinase was below the normal level but could be raised to the normal level by adding the ATP-trapping system. Thyroxine exerted a marked uncoupling effect on the oxidative phosphorylation of the fresh hepatoma in contrast to that of the liver mitochondria. EDTA counteracted the uncoupling by thyroxine of the oxidative phosphorylation of the hepatoma mitochondria. The conditions necessary for obtaining fatty acid oxidation, apart from being dependent upon the age of the transplants from which the mitochondria were prepared, were more exacting in the case of the hepatoma than of the liver mitochondria.

3. From the spontaneous activation of the ATPases, the release of DPN, the lowered P : O ratios, the effect of thyroxine on oxidative phosphorylation and the conditions necessary for fatty acid oxidation, it was concluded that the isolated BY 252 rat hepatoma mitochondria were more labile *in vitro* than rat liver mitochondria.

4. Similar studies made with mitochondria from 3 transplanted mouse hepatoma strains and many primary rat and mouse hepatomas induced by DAB and AAT, respectively, revealed also the labile character of these hepatoma mitochondria. In regard to certain enzymic properties some hepatoma particles were less labile than others. However, the labile character was not an exclusive property of isolated mitochondria from neoplastic tissues since liver mitochondria from a partly inbred guinea pig strain gave also evidence of an increased lability in comparison to mouse and rat liver mitochondria.

5. A parallel biological and biochemical study of livers from rats fed with DAB was made. Mitochondria from pre-neoplastic livers behaved like normal liver mitochondria except that the free ATPase was sometimes (somewhat) enhanced and that DNP had a smaller effect in those cases in which the pathological changes were very pronounced. Non-malignant local hyperplasias (" hyperplastomas ") solid trabecular hepatomas and less differentiated solid hepatomas, next to adeno and mixed solid-adeno hepatomas were encountered. Of the mitochondria isolated from these types of growth, those of the former two bore more resemblance to normal liver mitochondria than the latter. The mitochondria of pre-neoplastic mouse livers gave evidence of a greater lability than those of pre-neoplastic rat liver mitochondria.

6. The implications of the present findings are discussed. It is concluded that—compared with normal rat liver mitochondria—the decreased function of the isolated hepatoma mitochondria manifests itself as a result of the *in vitro* techniques and that the labile character, as well as the diminished content of certain enzymes, may be correlated with the particular fine-structural organization of the latter particles *in situ,* as suggested by electron microscopical examination.

Our sincere thanks are due to Glaxo Laboratories Ltd. for a gift of dextran, and to Professor Th. G. van Rijssel and Dr. J. C. H. de Man for permission to quote from the electron microscopical studies prior to publication.

### REFERENCES

ALLARD, C., DE LAMIRANDE, G. AND CANTERO, A.—(1957) *Cancer Res.*, **17**, 862.
BARKER, S. B. AND SUMMERSON, W. H. (1941) *J. biol. Chem.*, **138**, 535.
BERNHARD, W.—(1957) *Klin. Wschr.*, **35**, 251.—(1958) *Cancer Res.*, **18**, 491.
BIRBECK, M. S. C. AND REID, E.—(1956) *J. biochem. biophys. Cytol.*, **2**, 609.
CLERICI, E. AND CUDKOWICZ, G.—(1956) *J. nat. Cancer Inst.*, **16**, 1459.
CRABTREE, H. C.—(1929) *Biochem. J.*, **23**, 536.
DE MAN, J. C. H., BRAAMS, W. G., WEILAND, H. TH. and EMMELOT, P.—(1959) *Ned. Tijdschr. Geneesk.* **103**, 108.
DIANZANI, D. U.—(1954) *Biochim. biophys. Acta*, **14**, 514.—(1955) *Ibid.*, **17**, 391.
EMMELOT, P.—(1954) *Jaarb. Kankeronderzoek en bestrijding Ned.*, **4**, 75.—(1957*a*) *Biochem. biophys. Acta*, **23**, 668.—(1957*b*) *Exp. Cell Res.*, **13**, 601.
*Idem* AND BOS, C. J.—(1955) *Rec. Trav. chim. Pays-Bas*, **74**, 1343.—(1957*a*) *Enzymologia*, **18**, 179.—(1957*b*) *Exp. Cell. Res.*, **12**, 191.—(1957*c*) *Brit. J. Cancer*, **11**, 148.—(1958) *Exp. Cell Res.*, **14**, 132.
*Idem*, BOS, C. J. AND BROMBACHER, P. J.—(1956) *Brit. J. Cancer*, **10**, 188.
*Idem* AND BROMBACHER, P. J.—(1956) *Biochim. biophys. Acta* **22**, 487.—(1957) *Ibid.*, **23**, 435.
*Idem* AND VAN VALS, G. H.—(1957*a*) *Z. Krebsforsch.*, **62**, 116.—(1957*b*) *Ibid.*, **62**, 63.
ERNSTER, L.—(1956) *Exp. Cell Res.*, **10**, 704, 721.
FISKE, C. H. AND SUBBAROW, Y.—(1929) *J. biol. Chem.*, **81**, 629.
FORSSBERG, A. AND RÉVÉSZ, L.—(1957) *Biochim. biophys. Acta*, **25**, 165.
GLOCK, G. E. AND McLEAN, P.—(1957) *Biochem. J.*, **65**, 413.
GREENSTEIN, J. P.—(1953) "Biochemistry of Cancer", New York (Academic Press Inc.) 2nd edn.—(1956) *Cancer Res.*, **16**, 641.
HOCH, F. L. AND LIPMANN, F.—(1954) *Proc. nat. Acad. Sci. Wash.*, **40**, 909.
HOGEBOOM, G. H. AND SCHNEIDER, W. C.—(1953) *J. biol. Chem.*, **204**, 333.
HOWATSON, A. F. AND HAM, A. W.—(1955) *Cancer Res.*, **15**, 62.—(1957) *Canadian Cancer Conference*, **2**, 17.
HUNTER, JR., F. E.—(1956) *Int. Congr. Biochem., Brussels*, 1955, **3**, 298.
JOHNSON, M. S.—(1941) *Science*, **94**, 200.
KIELLEY, R. K.—(1952) *Cancer Res.*, **12**, 124.—(1957) *J. biol. Chem.*, **227**, 91.
KIELLEY, W. W. AND KIELLEY, R. K.—(1951) *Ibid.*, **191**, 485.
KIT, S. AND GRIFFIN, A. C.—(1958) *Cancer Res.*, **18**, 621.
KLEIN, G.—(1956) *Ann. N.Y. Acad. Sci.*, **63**, 640.
KLEMPERER, H. G.—(1955) *Biochem. J.*, **60**, 122.
LYNEN, F.—(1941) *Liebigs Ann.*, **564**, 120.—(1958) "Neuere Ergebnisse Chemie u. Stoffwechsel Kohlenhydrate", Berlin (Springer Verlag) p. 155.
McBURNEY, R. P., WOOLNER, L. B. AND WOLLAEGER, E. E.—(1950) *Proc. Mayo Clin.*, **25**, 606.
MARTIUS, C. AND HESS, B.—(1955) *Biochem. Z.*, **191**, 326.
MEDES, G., PADEN, G. AND WEINHOUSE, S.—(1957) *Cancer Res.*, **17**, 127.
NOVIKOFF, A. B.—(1957) *Ibid.*, **17**, 1010.
OLSON, R. E.—(1951) *Ibid.*, **11**, 571.
PARK, J. H., MERIWETHER, B. P., PARK, C. R., MUDD, S. M. AND LIPMANN, F.—(1956) *Biochim. biophys. Acta*. **22**, 403.
PERSKE, W. F., PARKS, JR., R. E. AND WALKER, D. L.—(1957) *Science* **125**, 1290.
POTTER, V. R., SIEKEVITZ, P. AND SIMONSON, H. C.—(1953) *J. biol. Chem.*, **205**, 893.
PURDOM, L., AMBROSE, E. J. AND KLEIN, G.—(1958) *Nature, Lond.*, **181**, 1586.
RACKER, E.—(1956) *Ann. N.Y. Acad. Sci.*, **63**, 1017.
REID, E. AND O'NEIL, M. A.—(1956) *Brit. J. Cancer*, **10**, 287.
REYNAFARJE, B. AND POTTER, V. R.—(1957) *Cancer Res.*, **17**, 1112.
SCHNEIDER, W. C. AND HOGEBOOM, G. H.—(1950) *J. nat. Cancer Inst.*, **10**, 969.

SIEKEVITZ, P., LÖW, H., ERNSTER, L. AND LINDBERG, O.—(1958) *Biochim. biophys. Acta*, **29**, 378.
SLATER, E. C. AND CLELAND, K. W.—(1953) *Biochem. J.*, **55**, 566.
SYLVÉN, B. AND MALMGREN, H.—(1957) *Acta radiol., Stockh.*, Suppl. 124.
TAPLEY, D. F., COOPER, C. AND LEHNINGER, A. L.—(1955) *Biochim. biophys. Acta*, **18**, 597.
WARBURG, O.—(1956) *Science*, **123**, 309.
WEBER, G. AND CANTERO, A.—(1957) *Cancer Res.* **17**, 995.
WENNER, C. E. AND WEINHOUSE, S.—(1953) *Ibid.*, **13**, 21.

### ADDENDUM

Since this paper went to press it has been reported by M. Birns, E. Essner and A. B. Novikoff (1959, *Proc. Amer. Ass. Cancer Res.*, **3**, 7) that, contrary to the finding of Allard *et al.* (1957) the glutamic acid dehydrogenase was present in the Novikoff rat hepatoma kept in the laboratory of the former authors. A study of the Dunning transplantable rat hepatoma LC 18/81A (Pitot, N. C., Fohn, C. H., Clark, Jr., W. H. and Farber, E., 1959, *Proc. Amer. Ass. Cancer Res.*, **3**, 52) has led to the same general conclusion as that reached in the present paper, namely " that a number of different hepatomas must be studied before it can be assumed that any particular set of biochemical or morphological peculiarities observed in one hepatoma is characteristic and distinctive for hepatic neoplasms as a group." In this connection it might be of interest to investigate whether the decrease in or the absence of certain enzymic activities are real or due to endogenous inhibitors.